# Ethnobotany, Phytochemistry, and Pharmacological Activities of *Ocimum* Species in Low- and Middle-Income Countries: A Systematic Review

**DOI:** 10.3390/ijms27125540

**Published:** 2026-06-18

**Authors:** Chikondi Maluwa, Blecious Zinan’dala, Hataichanok Chuljerm, Wason Parklak, Kanokwan Kulprachakarn

**Affiliations:** 1 School of Health Sciences Research, Research Institute for Health Sciences, Chiang Mai University, Chiang Mai 50200, Thailand; chikondi_maluwa@cmu.ac.th (C.M.); dysonblecious_zina@cmu.ac.th (B.Z.); hataichanok.ch@cmu.ac.th (H.C.); wason.p@cmu.ac.th (W.P.); 2 Department of Clinical Sciences, Malawi College of Health Sciences, Blantyre Campus, Blantyre Private Bag 396, Malawi; 3 Department of Clinical Sciences, Malawi College of Health Sciences, Lilongwe Campus, Lilongwe P.O. Box 30368, Malawi

**Keywords:** *Ocimum*, basil, ethnopharmacology, phytochemistry, essential oils, pharmacological activity, traditional medicine, systematic review, low- and middle-income countries

## Abstract

*Ocimum* species (family Lamiaceae) are among the most extensively utilized medicinal plants across low- and middle-income countries (LMICs), yet their pharmacological evidence base has not been comprehensively synthesized within an LMIC healthcare framework. A systematic review was conducted following PRISMA 2020 guidelines and a prospectively registered protocol (PROSPERO). Five electronic databases, PubMed, Scopus, Embase, Web of Science, and Google Scholar, were searched from January 2010 to December 2025. Studies reporting ethnobotanical, phytochemical, or pharmacological data on any *Ocimum* species were eligible. The study selection, quality assessment and data extraction were done by two independent reviewers utilizing Rayyan software. Findings were synthesized using a narrative approach. Ninety-seven studies were included. *O. basilicum*, *O. tenuiflorum*, and *O. gratissimum* were most studied. Key bioactive constituents rosmarinic acid, eugenol, linalool, β-caryophyllene, and ursolic acid, demonstrated consistent antimicrobial [minimum inhibitory concentration (MIC): 0.31–1.25 mg/mL], antioxidant [2,2-diphenyl-1-picrylhydrazyl (DPPH) IC_50_: 12.5–89.3 µg/mL], anti-inflammatory (35–55% edema reduction), and antidiabetic (α-glucosidase IC_50_: 0.3–1.5 mg/mL) activities. Larvicidal efficacy exceeding 90% against *Anopheles* spp. was demonstrated in field trials. The safety profile was broadly favorable (LD_50_ > 5000 mg/kg). *Ocimum* species represent a pharmacologically credible and preclinically well-supported botanical resource with practical relevance for LMIC health systems, particularly in antimicrobial, antidiabetic, anti-inflammatory, and vector-control applications. To realize their therapeutic potential, future research must prioritize LMIC-contextualized randomized controlled trials, standardized phytochemical reporting, and chemotype-aware product development.

## 1. Introduction

Indigenous plant knowledge represents one of humanity’s oldest and most geographically distributed pharmacopeias, yet it remains systematically marginalized within evidence-based medicine frameworks. In low- and middle-income countries (LMICs) defined by the World Bank as nations with gross national income (GNI) per capita below US$12,535 [1], primary healthcare access remaining constrained by human resources, infrastructural deficits, medication cost, and geographic isolation [2,3,4]. In this context, traditional plant medicine constitutes a functional healthcare system for an estimated 80% of rural populations across sub-Saharan Africa, South and Southeast Asia, and Latin America [5,6,7,8].

Among the most ecologically widespread and ethnobotanically significant plant genera in these regions is *Ocimum* L. (family Lamiaceae), collectively referred to as basil. The genus *Ocimum* encompasses approximately 65 recognized species [9,10], of which *O. basilicum* L. (sweet basil) is perhaps the most globally recognized, used in Mediterranean and Southeast Asian culinary traditions, and commercially cultivated in countries including Egypt, India, Indonesia, and Nigeria [11,12].

However, in some LMIC contexts the more therapeutically important species are *O. tenuiflorum* L. (holy basil or tulsi), mostly used in Hindu Ayurvedic medicine as an adaptogen and immunomodulator [13]; *O. gratissimum* L. (African basil, scent leaf or clove basil), extensively used in Ghanaian, Nigerian, and Kenyan ethnomedicine for infectious and metabolic conditions [14,15]; *O. americanum* (American basil, hairy basil), mostly used in respiratory applications, management of constipation, and other infections [16]; *O. kilimandscharicum* (Kilimanjaro camphor basil, African Blue basil), and *O. canum* Sims (Malawi camphor basil), mostly used as mosquito repellent and for treating infections [17,18]. These regional species share substantial phytochemical overlaps with *O. basilicum* L.

Basil holds deep-rooted significance across multiple healing traditions. It has been prescribed for digestive disorders, respiratory ailments, and stress-related conditions [19,20,21]. Traditional Chinese Medicine employs basil preparations to regulate energy flow, relieve pain, and treat skin conditions [22,23]. In Unani medicine it is used as a carminative, diuretic, and anti-inflammatory agent [24,25]. Across Sub-Saharan Africa, traditional healers deploy basil species against malaria, asthma, headache, wound infections, and gastrointestinal disorders [9,10,26,27].

Basil is widely utilized across low- and middle-income countries for culinary, cultural, and medicinal purposes, yet the evidence base supporting its therapeutic applications remains fragmented and inconsistently synthesized. While individual studies have reported diverse bioactive compounds, such as essential oils, flavonoids, and phenolic acids, with several health benefits [12,25,28,29,30,31,32,33], there is limited integration of ethnobotanical knowledge with phytochemical and pharmacological findings. This gap restricts the translation of traditional practices into evidence-based and locally appropriate health interventions. Moreover, LMICs face a disproportionate burden of noncommunicable and infectious diseases, alongside constrained access to conventional healthcare [34], highlighting the need to explore affordable, plant-based alternatives.

This systematic review therefore consolidates existing evidence on the ethnobotany, phytochemistry, and pharmacological potential of *Ocimum* species, identifies research gaps, and informs future studies and the development of sustainable phytotherapeutics tailored to resource-limited settings.

### 1.1. Botanical Description and Taxonomic Considerations

#### 1.1.1. Genus Overview

**Figure 1:**
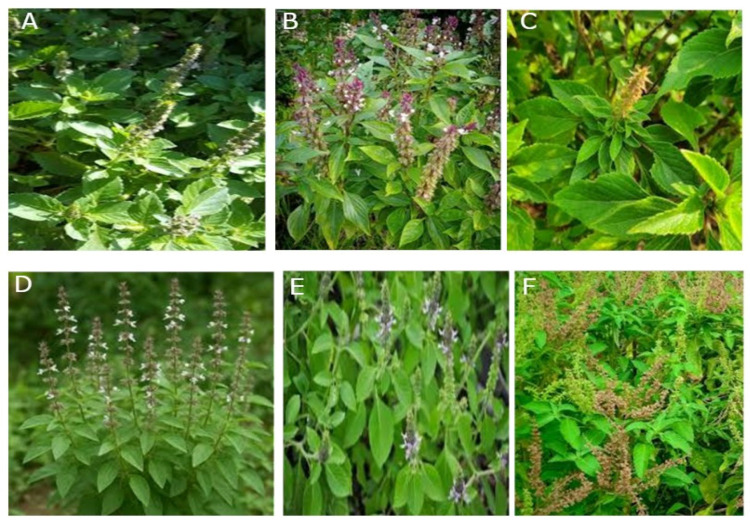
A representative picture of different *Ocimum* Species. (**A**) *O. tenuiflorum*; (**B**) *O. basilicum L.* (**C**) *O. gratissimum* L.; (**D**) *O. canum* Sims; (**E**) *O. americanum* L.; (**F**) *O. kilimandscharicum* [35,44].

Basil is an erect, much-branched annual herb native to tropical regions of Asia and Africa (Figure 1). The plant has been cultivated for more than 5000 years and is today grown across all inhabited continents. The genus *Ocimum* L. belongs to the tribe *Ocimeae*, subfamily *Nepetoideae*, within the family Lamiaceae [9,10,35,36,37,38]. The genus is morphologically and phylogenetically diverse, comprising approximately 65 accepted species, with centers of diversity concentrated in tropical Africa and Asia, geographic zones with the world’s highest health burden [9,10]. Morphologically, basil plants grow 30–60 cm in height and are characterized by aromatic annual or perennial herbs and shrubs, typically bearing opposite, stalked leaves with glandular trichomes that secrete essential oils. The flowers grow in little circles around the stem, and each flower has two lips. The fruit has four small, smooth seeds. Phylogenetically, *Ocimum* represents a taxonomically challenging group [35]. New studies of DNA showed that some plants which were thought to be different are actually the same. For example, the incorporation of *O. sanctum* L. into *O. tenuiflorum* L. [39,40,41,42,43].

#### 1.1.2. Species of Major LMIC Relevance

Table 1 summarizes the *Ocimum* species identified in this review as medicinally significant within LMIC contexts, with their primary geographic distribution, common names, and predominant traditional use.

## 2. Materials and Methods

### 2.1. Protocol and Registration

This systematic review was conducted and reported in accordance with the Preferred Reporting Items for Systematic Reviews and Meta-Analyses (PRISMA 2020) guidelines [57]. The review protocol was registered in the International Prospective Register of Systematic Reviews (PROSPERO; registration number: CRD42026138620), ensuring methodological transparency and minimizing the risk of reporting bias.

### 2.2. Search Strategy

A comprehensive and systematic literature search was performed across five electronic databases including PubMed, Scopus, Embase, Web of Science, and Google Scholar. The search was conducted on 22 March 2026 by two independent reviewers. Complete Boolean search strings for each database and language restrictions are provided in full in Supplementary Table S1.

The strategy combined controlled vocabulary (Medical Subject Headings [MeSH], where applicable) with free-text keywords related to *Ocimum* L. and its pharmacological activities. Boolean operators (“AND” and “OR”) were applied to optimize sensitivity and specificity. The core search string included terms such as “*Ocimum* L.” OR “basil,” combined with pharmacological descriptors including “pharmacology,” “bioactivity,” “therapeutic,” “antioxidant,” “antimicrobial,” “anti-inflammatory,” “anticancer,” “antidiabetic,” “neuroprotective,” and “cardioprotective,” alongside terms describing plant derivatives such as “extract,” “essential oil,” “phytochemical,” “compound,” and “constituent.” The search was restricted to studies published in English between 1 January 2010, and 31 December 2025. For Google Scholar, the first 300 records (30 pages) per discrete query were screened, consistent with established gray-literature supplementation practice for systematic reviews [57]. To ensure completeness, reference lists of included articles and relevant reviews were manually screened to identify additional eligible studies.

### 2.3. Eligibility Criteria

Studies were eligible if they evaluated the pharmacological activities of *Ocimum* extracts, essential oils, or isolated compounds using in vitro, in vivo (animal), or clinical (human) designs and reported quantitative outcomes (e.g., IC_50_, MIC, percentage inhibition, or effect size). Only peer-reviewed original research articles published between January 2010, and December 2025 were included. Studies focusing exclusively on agricultural, horticultural, or food science applications without pharmacological endpoints were excluded, as were conference abstracts, editorials, letters, those lacking sufficient methodological detail, duplicate publications, and secondary reports of previously published datasets.

Stream-specific eligibility criteria were applied. Ethnobotanical studies were required to document traditional *Ocimum* use in low- and middle-income countries (LMICs) using primary data collection, with species identified at least to the genus level. Phytochemical studies were eligible if they identified or quantified compounds using validated analytical techniques, including GC–MS, LC–MS, HPLC–DAD, or equivalent methods. In vitro studies had to report quantitative pharmacological outcomes, while in vivo studies were required to employ defined animal models and report measurable pharmacological endpoints. Clinical studies included controlled trials and observational designs reporting pharmacological outcomes. Field and vector-control studies were included when they evaluated outcomes such as larval mortality or repellent efficacy under field or semi-field conditions.

LMIC relevance was operationalized as satisfaction of at least one of the following criteria: (1) the study was conducted in a country classified as low- or middle-income by the World Bank (2024–2025 classification) [1]; (2) the plant material was obtained from a LMIC country; (3) the traditional use documented was from an LMIC ethnomedicinal system; (4) the disease domain addressed is of high epidemiological burden in LMICs; or (5) the species investigated is geographically accessible and agriculturally available in LMICs. Studies from high-income countries were included where one or more of criteria (2–5) were met. Review articles were excluded from the primary evidence synthesis but were used for background context and reference-list screening.

### 2.4. Study Selection

All retrieved records were initially imported into EndNote Version 21 for duplicate identification and removal before being transferred to Rayyan systematic review software for screening. An additional automated deduplication process was conducted in Rayyan, followed by manual verification to ensure accurate identification and removal of duplicate records. Titles and abstracts were independently screened by two independent reviewers based on the predefined eligibility criteria, followed by full-text assessment of potentially relevant articles. Disagreements at any stage were resolved through discussion and consensus, with a third reviewer consulted where necessary.

### 2.5. Data Extraction

A standardized data extraction form was used to ensure consistency and reproducibility. Data extracted from each included study encompassed study characteristics such as authorship, year of publication, country, World Bank income classification and study design; detailed information on plant material including species verification, plant part used, geographical origin, and extraction method; phytochemical composition including identified compounds and analytical techniques; experimental models and assay parameters; pharmacological activities assessed along with quantitative outcomes such as IC_50_, MIC, and inhibition rates; as well as safety, toxicity, and relevant methodological quality indicators. Data extraction was performed independently by two reviewers, and any discrepancies were resolved through discussion and consensus. Full extraction data are provided in Supplementary Table S2.

### 2.6. Quality Assessment

The methodological quality and risk of bias of included studies were evaluated using validated tools appropriate to each study design. In vitro studies were assessed using a modified CONSORT checklist for preclinical research, animal studies were evaluated using SYRCLE’s Risk of Bias tool, clinical studies were assessed using the Cochrane Risk of Bias tool version 2 (RoB 2), and Joanna Briggs Institute (JBI) critical appraisal for ethnobotanical surveys [58,59,60]. All assessments were conducted independently by two reviewers, with disagreements resolved through discussion to ensure consistency and reliability.

### 2.7. Data Synthesis

Given the substantial heterogeneity across included studies in terms of experimental design, plant material preparation, extraction techniques, solvent systems, chemotype variation, dosing regimens, and outcome measures, a quantitative meta-analysis was not feasible. Consequently, findings were synthesized using a narrative approach, with results organized according to pharmacological activity domains such as antioxidant, antimicrobial, and anti-inflammatory effects. Where appropriate, semi-quantitative comparisons, including IC_50_ and MIC values, were tabulated to facilitate contextualization of findings.

Cross-study numerical comparisons were not possible hence, IC_50_ or MIC values derived from different extract types, assay platforms, and solvent concentrations are not directly comparable. All numerical cross-study comparisons are contextualized by specifying the extract type, species, assay model, and study context. Drug-equivalence statements are restricted to within-study comparisons from the original publications. Risk-of-bias findings were integrated into domain-level certainty assessments.

## 3. Results

### 3.1. Study Selection and Study Characteristics

**Figure 2:**
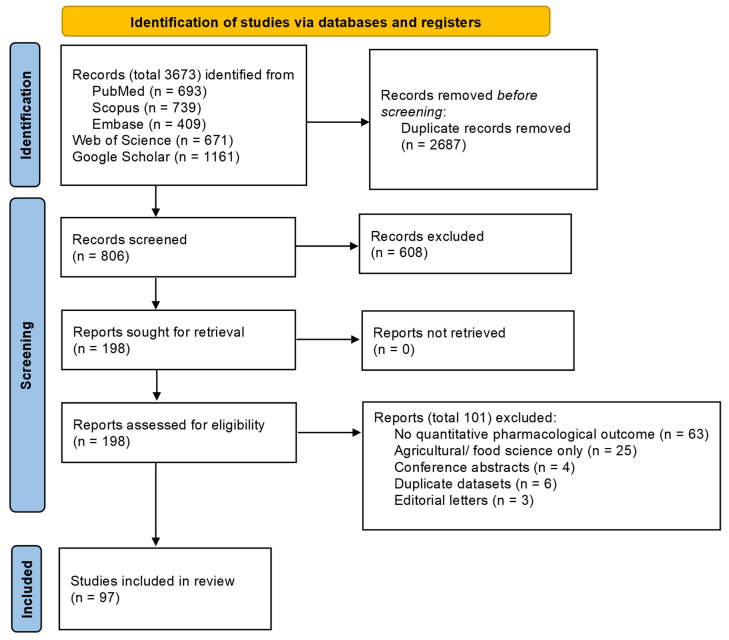
PRISMA flow diagram.

The study selection process is illustrated in the PRISMA flow diagram (Figure 2). A total of 3673 records were identified from five databases (PubMed, Scopus, Embase, Web of Science, and Google Scholar). After removing 2867 duplicates, 806 unique records remained for screening. Following title and abstract review, 608 records were excluded. Of the 198 full-text articles assessed for eligibility, 97 met the inclusion criteria and were included in the review.

The 97 included studies were predominantly preclinical, comprising in vitro investigations (n = 47) and animal-based in vivo experiments (n = 39). Only two randomized controlled trials and three dedicated toxicological studies were identified. Additionally, nine ethnobotanical surveys contributed to the evidence base, with field/vector-control and some studies overlapping multiple evidence streams. Studies spanned twelve pharmacological domains, examined six *Ocimum* species, and were conducted across Africa, Asia, and the Americas, confirming a geographically distributed yet predominantly preclinical evidence base. Of the 97 studies, 81 (84%) originated from LMICs or used LMIC-origin plant material. The geographic distribution and study characteristics of all included studies are summarized in Supplementary Table S2.

**Figure 3:**
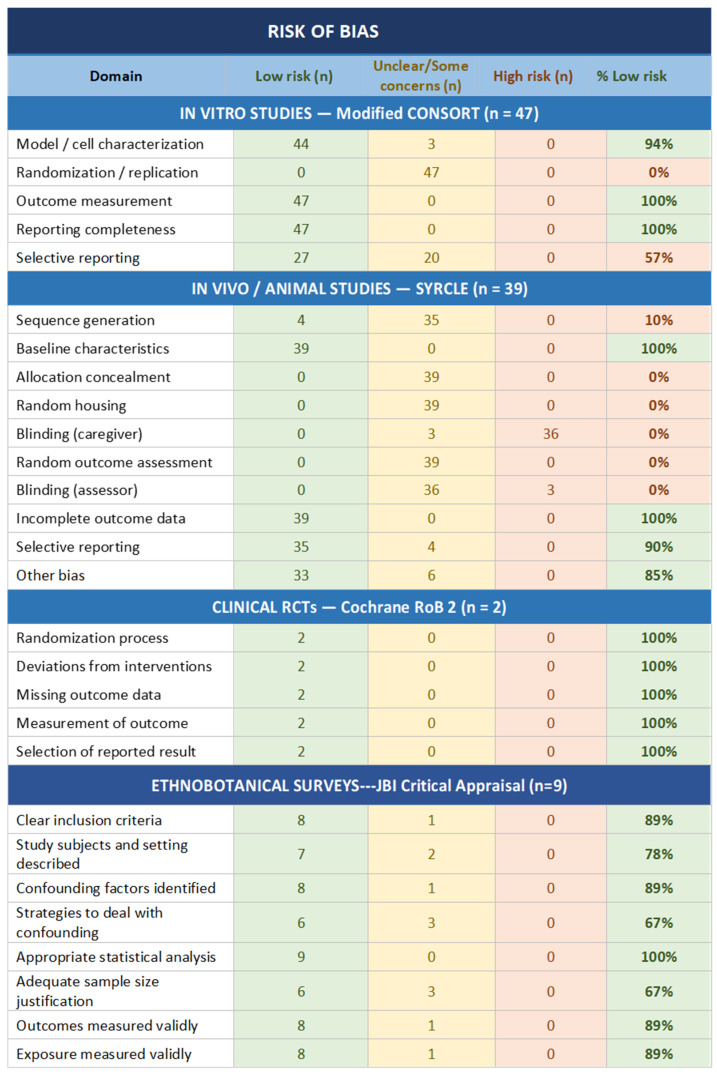
Summary of risk-of-bias assessment. **Note:** n: number of studies; JBI: Joanna Briggs Institute; RoB: risk of bias; Low risk: item adequately addressed, low likelihood of bias; Unclear/Some concerns: item partially addressed or unreported, uncertain bias; High risk: item not addressed or evident methodological flaw.

All the included studies were subjected to risk-of-bias assessment across domains, for in vitro studies (Modified CONSORT, n = 47), in vivo/animal studies (SYRCLE, n = 39), clinical randomized controlled trials (Cochrane RoB 2, n = 2), and ethnobotanical surveys (JBI critical appraisal, n = 9). Risk-of-bias assessment revealed a heterogeneous methodological quality profile. While many in vitro studies showed adequate reporting of assay conditions and positive controls, the majority of animal studies demonstrated unclear or high risk of bias in key SYRCLE domains, including random housing, allocation concealment, caregiver blinding, outcome-assessor blinding, and selective outcome reporting. The two included clinical trials and nine ethnobotanical surveys demonstrated low risk of bias across all Cochrane RoB 2 domains and the JBI critical appraisal domain respectively. The overall assessment suggests an acceptable level of methodological quality, though some studies have some concerns of high risk of bias in some areas. Figure 3 shows a summary of risk of bias across the included studies.

### 3.2. Phytochemical Composition

#### 3.2.1. Essential Oil Constituents

The most extensively characterized phytochemical fraction in *Ocimum* species is the essential oil (EO), obtained predominantly by hydrodistillation from aerial parts, with yield varying from 0.1% to 1.8% (*w*/*w*) depending on species, chemotype, developmental stage, geographic origin, and extraction conditions [31,61,62,63,64,65]. Gas chromatography–mass spectrometry (GC-MS) profiling has enabled detailed characterization of over 150 volatile compounds across the genus. The main essential oils found in important basil plants in low- and middle-income countries include linalool, eugenol, estragole, camphor, 1,8-cineole, and methyl chavicol (Table 2).

In *O. basilicum*, the dominant chemotypes identified in tropical Africa and Asia are the linalool, the methyl chavicol (estragole), and the eugenol, with distribution patterns strongly correlated with geographic origin [66,67,68]. Cultivars of *O. basilicum* are characterized by high linalool content (35–65%) and higher methyl chavicol concentrations (30–45%) [39,63,69,70].

For *O. tenuiflorum*, molecular studies have documented eugenol as the primary constituent (up to 71%), accompanied by β-caryophyllene, methyl eugenol, and germacrene-D [13,71,72]. Interestingly, some cultivars of the same species exhibit a methyl eugenol-dominant chemotype [47,71,73]. This intraspecific chemotypic variation indicates that phytochemical composition is not species-invariant but is substantially influenced by cultivar, geographic origin, and environmental conditions, a critical consideration for pharmacological interpretation and product standardization.

Eugenol is the essential oil that is dominant in *O. gratissimum* ranging from 60 to 85%. It is widely regarded as the primary bioactive compound responsible for the species’ strong antimicrobial properties [74,75,76,77]. Akintunde et al. (2025) demonstrated that *O. gratissimum* leaves extracts exhibited significant antimicrobial activity against both Gram-positive and Gram-negative bacteria, with the highest inhibition observed against *Staphylococcus aureus* and *Pseudomonas aeruginosa* [78].

*O. kilimandscharicum*, contains camphor (40–65%) and 1,8-cineole (15–30%) as the characterized chemotype, which are consistent with its traditional use in respiratory conditions and as a vector control agent [79,80,81]. Similarly, *O. americanum* L. contains 1.8-cineole (51.8%) and camphor (17.4%) as the major constituents of the EO [82,83,84]. Consistent with its use in respiratory conditions and vector control, it also demonstrates antibacterial activity against Gram-positive bacteria and fungi [16,85,86,87]. *O. canum* Sims has high levels of carvacrol, p-Cymene, camphor. It has been used as an antidiabetic, antimicrobial, mosquito repellent, antipyretic, for colds, and respiratory related complaints [50,51,52,88,89].

#### 3.2.2. Other Phytoconstituents

Beyond the EO fraction, *Ocimum* species accumulate an array of non-volatile metabolites of significant pharmacological relevance (Table 2). Phenolic acids, particularly rosmarinic acid and caffeic acid, are among the most consistently detected compounds in polar extracts across multiple species. Rosmarinic acid has been identified as a principal constituent contributing to antioxidant activity in *O. basilicum*, *O. tenuiflorum*, and *O. americanum* [68,71,72,90]. Quantification studies using HPLC-DAD have reported rosmarinic acid concentrations of 2–15 mg/g dry weight in leaf extracts of basil from Egyptian, Indian, and Thai accessions [91].

Flavonoids constitute another major class of non-volatile bioactive compounds in basil. Apigenin, luteolin, quercetin, vicenin-2, and orientin have been identified across multiple species using liquid chromatography mass spectrometry (LC-MS) techniques [9,31,92]. Notably, orientin and vicenin-2, two C-glycosyl flavones commonly identified in several *Ocimum* species, have demonstrated significant radioprotective and immunomodulatory properties in preclinical investigations, suggesting their potential role in mitigating radiation-induced cellular damage while modulating immune responses [93,94]; although, clinical validation of these specific effects is currently lacking.

Triterpenes, specifically ursolic acid and oleanolic acid, have been isolated from basil and are considered mechanistically important for observed anti-inflammatory and hepatoprotective effects [9,95,96]. Studies have isolated ursolic acid from basil leaf extracts and documented inhibitory activity against cyclooxygenase-2 (COX-2) [97,98,99,100], a preclinical finding with direct translational relevance for affordable anti-inflammatory interventions in LMICs where nonsteroidal anti-inflammatory drugs (NSAIDs) impose significant cost burdens.

**Table 2:** Phytochemical composition of *Ocimum* species.

Species	Chemical Class	Phytochemical Compound(s)	Analytical Method	Primary Bioactivity	References
***Ocimum*** ***basilicum* L.**	Monoterpene Phenylpropanoid Phenolic acid Flavonoid	Linalool (35–65%) Methyl chavicol (estragole) (30–45%) Eugenol (71%) Rosmarinic acid Quercetin Rutin Apigenin	GC-MS (EO); HPLC-DAD (phenolics)	Antimicrobial Anti-inflammatory Antioxidant Anticancer Cardioprotective	[12,32,33,62,64,86,88]
***Ocimum*** ***tenuiflorum* L.**	Monoterpene Sesquiterpene Pentacyclic triterpenoid Flavonoid Flavone-C-glycoside	Eugenol (71%) β-Caryophyllene (5–20%) Methyl eugenol Ursolic acid Oleanolic acid Luteolin Orientin Vicenin Apigenin-7-O-glucuronide	GC-MS (EO); HPLC-DAD (phenolics, flavonoids); LC-MS/MS (non-volatile fraction)	Antimicrobial Anti-inflammatory Analgesic Antidiabetic Hepatoprotective Anticancer Neuroprotective Antioxidant	[13,47,67,68,88,91,93]
***Ocimum*** ***gratissimum* L.**	Monoterpene Phenylpropanoid Alkaloid Saponin Tannin Flavonoid	Eugenol (dominant; ~60–85% EO) Thymol (5–65%) γ-Terpinene (5–15%) p-Cymene (3–10%) Alkaloids Saponins Tannins Flavonoid-rich fractions	GC-MS (EO); HPLC-UV (phenolics)	Antimicrobial Antifungal Anti-inflammatory Immunomodulatory	[14,15,48,70,71,73,74]
***Ocimum*** ***americanum* L.**	Bicyclic monoterpene Cyclic monoterpene ether	Camphor (17.4%) 1,8-Cineole (eucalyptol) (51.8%) Linalool (5–15%)	GC-MS; GC-FID (Quantification)	Antimicrobial Antifungal Respiratory relief	[16,78,79,81,83]
***Ocimum canum* Sims**	Monoterpene Monoterpenoid phenol Cymene derivative	Carvacrol (20–40%) p-Cymene (10–25%) Camphor (8–20%) γ-Terpinene (0.03–20%)	GC-MS; HPLC-DAD (phenolics)	Antimicrobial Antifungal Antidiabetic Antipyretic Larvicidal/Mosquito repellent	[50,51,52,84,85]
***Ocimum*** ***kilimandscharicum***	Bicyclic monoterpene Monoterpene alcohol	Camphor (40–65%) α-Thujene (5–12%) β-Terpineol (15–30%)	GC-MS	Antimicrobial Antifungal Analgesic Larvicidal/Mosquito repellent	[17,53,54,75,76]

Note: EO: essential oil; GC-MS: gas chromatography–mass spectrometry; GC-FID: gas chromatography–flame ionization detector; HPLC-DAD: high-performance liquid chromatography with diode array detection; HPLC-UV: high-performance liquid chromatography with ultraviolet detection; LC-MS/MS: liquid chromatography–tandem mass spectrometry.

### 3.3. Pharmacological Activities of Basil

#### 3.3.1. Antimicrobial Activity

Antimicrobial properties constitute the most extensively investigated biological activity of *Ocimum* EOs and extracts in LMIC-focused literature. Across the evidence base (37 studies), in vitro antibacterial and antifungal assays, primarily employing disk diffusion and broth microdilution methods, consistently demonstrate broad-spectrum activity. Studies have reported minimum inhibitory concentrations (MICs) of 0.31–1.25 mg/mL for eugenol-rich *O. gratissimum* EO against *Salmonella typhimurium*, *Vibrio cholerae*, and *Staphylococcus aureus* [101,102], pathogens of high epidemiological relevance in sub-Saharan Africa. Similarly, Ajmal et al. (2025) showed that basil EOs significantly inhibited *Fusarium moniliforme* and *Aspergillus flavus*, highlighting its role in antifungal activity and preventing food from spoilage [103].

Studies have shown basil to have antimicrobial activity against different microbial species, with MICs ranging from 0.3 to 5 mg/mL for bacteria and 0.5–8 mg/mL for fungi, though notable heterogeneity exists due to variations in chemotype, extraction methods, and assay conditions [104,105] with basil EOs and extracts exhibited particularly broad-spectrum efficacy. Importantly, emerging evidence shows that among Gram-positive organisms, methicillin-resistant *Staphylococcus aureus* (MRSA) was consistently susceptible (MIC: 0.6–2.5 mg/mL), with eugenol and linalool identified as key bioactive compounds mediating membrane disruption and biofilm inhibition [106,107,108]. Activity against Gram-negative bacteria was comparatively moderate (E. coli MIC: 1.5–4 mg/mL and Pseudomonas aeruginosa MIC: 2–6 mg/mL), likely reflecting the protective role of the outer membrane [33,109]. Antifungal effects were also substantial, with activity against systemic candidiasis in mice (MIC: 0.5–2 mg/mL) like that of ketoconazole, *Aspergillus niger* (MIC: 1–3 mg/mL), and dermatophytes relevant to sub-Saharan disease burden [103,110]. Mechanistically, it has been demonstrated that methyl trans-cinnamate disrupts fungal ergosterol biosynthesis, providing a plausible biochemical basis for the traditional use of *Ocimum* species in managing skin and wound infections [111].

All antimicrobial conclusions are based on preclinical data. Substantial inter-study heterogeneity in MIC values, driven by chemotype variation, extraction method, solvent system, and assay forma, constrains cross-study numerical comparisons. Preclinical MIC values are not predictive of clinical efficacy; no clinical antimicrobial trial was identified, representing a critical translational gap.

#### 3.3.2. Antioxidant Activity

Antioxidant capacity represents one of the most extensively investigated pharmacological properties of *Ocimum* species and remains a dominant theme across the evidence base (30 studies). This activity has been evaluated using multiple complementary assays, including DPPH (2,2-diphenyl-1-picrylhydrazyl) radical scavenging, ABTS (2,2-azino-bis-3-ethylbenzothiazoline-6-sulfonic acid) decolorization, ferric reducing antioxidant power (FRAP), cupric reducing antioxidant capacity (CUPRAC), and lipid peroxidation markers such as thiobarbituric acid-reactive substance (TBARS) and malondialdehyde (MDA). Collectively, these assays demonstrate substantial radical-quenching capacity, with DPPH IC_50_ values ranging from 12.5 to 89.3 µg/mL, largely attributable to phenolic constituents, particularly rosmarinic acid and chicoric acid [32,33,112,113,114]. Consistently, hydroalcoholic and aqueous extracts outperform essential oils in antioxidant benchmarks, reflecting the higher abundance of polar phenolic compounds in these fractions [33]. The superior antioxidant activity of polar fractions likely arises from both their higher phenolic acid content and the intrinsically greater radical-scavenging efficiency of rosmarinic and caffeic acids, whose multiple aromatic hydroxyl groups promote hydrogen atom transfer and single-electron transfer mechanisms. Consequently, these compounds exhibit stronger antioxidant activity than the major essential oil monoterpenes (linalool, camphor, and 1,8-cineole) on a molar basis [112,114].

Comparative analyses further highlight the influence of geographical origin and chemotype. Studies have reported that tropical *O. basilicum* varieties exhibited significantly higher total phenolic content (TPC: 48.2 mg GAE/g DW) than temperate cultivars (28.7 mg GAE/g DW), with correspondingly lower DPPH IC_50_ values (12.4 vs. 24.6 µg/mL) [115], suggesting that LMIC-derived germplasm may constitute a more potent antioxidant resource than those from other regions. This enhanced phenolic accumulation is likely driven by environmental stressors such as elevated UV-B exposure, herbivory, and soil mineral variability, which upregulate phenylpropanoid pathway activity [116,117]. Supporting this, Wójciak et al. (2024) demonstrated that polyphenolic fractions account for approximately 60–70% of DPPH activity, reinforcing the central role of rosmarinic acid in the antioxidant activity [97].

Evidence from other studies further highlight the robustness of antioxidant effects, with significant inter-cultivar variability (DPPH IC_50_: 15.2–67.8 µg/mL) strongly correlated with total phenolic content (r^2^ = 0.89, *p* < 0.001), highlighting the importance of chemotype selection for functional applications [32,118,119]. Importantly, in vivo studies demonstrate biological relevance, where oral administration of basil extracts (200–400 mg/kg/day) significantly enhanced endogenous antioxidant defenses, including superoxide dismutase (SOD), catalase (CAT), glutathione peroxidase (GPx), and glutathione reductase (GR), while reducing hepatic and renal MDA levels in oxidative stress models [120,121,122]. Together, these preclinical findings position *Ocimum* species as a potent, yet underutilized, source of natural antioxidants with significant implications for managing oxidative stress-related conditions in LMIC contexts although human clinical evidence for meaningful antioxidant benefit at achievable doses is lacking.

#### 3.3.3. Anti-Inflammatory Activity

Anti-inflammatory activity was documented in 39 of the included studies, reflecting a substantial body of evidence derived from complementary in vitro and in vivo models [25,90]. In vitro investigations, primarily using LPS-stimulated RAW 264.7 macrophages, demonstrate that *Ocimum* extracts, particularly from *O. basilicum*, inhibit key inflammatory mediators, including nitric oxide (NO) production, cyclooxygenase enzymes (COX-1/COX-2; IC_50_: 25–80 µg/mL), and lipoxygenase (LOX; IC_50_: 15–60 µg/mL), alongside suppression of pro-inflammatory cytokines (TNF-α, IL-1β, IL-6) [84,90,93]. Mechanistically, these effects are largely mediated through inhibition of NF-κB p65 nuclear translocation, coupled with activation of the Nrf2/HO-1 pathway, which enhances cytoprotective and antioxidant responses [90,93,116]. Consistent with these findings, NF-κB suppression and pro-inflammatory cytokine downregulation have been identified as central molecular pathways, with eugenol, ursolic acid, and β-caryophyllene implicated as the principal bioactive constituents mediating these effects [90,94,95].

In vivo, anti-inflammatory efficacy has been consistently demonstrated in rodent models. Oral administration of *Ocimum* extracts (100–200 mg/kg) reduced carrageenan-induced paw edema by 35–55%, decreased cotton pellet granuloma formation by 35–50%, and significantly attenuated adjuvant-induced arthritis severity [84]. Oral administration of aqueous leaf extract of *O. tenuiflorum* at 200 mg/kg has been reported to reduce paw edema by a magnitude comparable to the standard NSAID indomethacin at 10 mg/kg, highlighting the pharmacological relevance of this species [13,47]. Additional experimental models, including croton oil-induced ear edema, further corroborate these anti-inflammatory effects [84,90]. Phytochemical analyses identify linalool and rosmarinic acid as dominant anti-inflammatory mediators, while eugenol contributes both anti-inflammatory and analgesic effects through modulation of TRPV1 receptors and opioid pathways [90,93,95].

These findings are of particular epidemiological significance in LMIC contexts, where inflammatory conditions span both infectious and non-communicable diseases [34]. The convergence of anti-inflammatory mechanisms observed across *Ocimum* species in preclinical studies suggests their potential as multifunctional therapeutic agents [25,33,90]; however, clinical validation in LMIC populations remains a critical evidence gap. Additionally, anti-inflammatory animal evidence is substantially weakened by the risk-of-bias profile: randomization, allocation concealment, and blinding were absent in the majority of SYRCLE-assessed studies, likely inflating reported effect sizes. Drug comparisons with indomethacin are within-study parallels only and do not imply clinical equivalence.

#### 3.3.4. Antidiabetic Activity

Thirty-two of the included studies have documented antidiabetic activity in *Ocimum* species, reflecting growing research attention in response to the escalating burden of type 2 diabetes mellitus (T2DM) in LMICs, where approximately 80% of global cases are concentrated [123]. Within this evidence base, *O. tenuiflorum* has shown that 250–500 mg/kg of hydroalcoholic leaf extract orally for 21 days in diabetic rats yielded the same results as glibenclamide [124]. Ogidi et al. (2024) [125] reported that 400 mg/kg of leaf extract per day had comparable results as taking 70 mg/kg of metformin in diabetic albino rats. This intervention resulted in significant reductions in fasting blood glucose, total cholesterol, triglycerides, low density lipoprotein and increase in high density lipoprotein [125]. Complementary mechanistic studies identified potent α-amylase (IC_50_ = 32.4 μg/mL) and α-glucosidase (IC_50_ = 18.9 μg/mL) inhibition, largely attributed to rosmarinic acid and caffeic acid [126,127], suggesting a feasible, low-cost diabetic management given its abundance in widely accessible basil species.

In vitro assays further confirmed inhibition of carbohydrate-digesting enzymes, with α-glucosidase IC_50_ values ranging from 0.3 to 1.5 mg/mL and α-amylase IC_50_ values from 0.5 to 2 mg/mL, demonstrating significant inhibition activity [128]. Moderate dipeptidyl peptidase-IV (DPP-IV) inhibition (20–40% at 100 µg/mL) has also been reported, indicating potential incretin-mediated effects [129]. In vivo evidence from streptozotocin-induced diabetic rat models shows that basil aqueous and ethanolic extracts (200–400 mg/kg/day, orally) reduce fasting blood glucose by 25–40%, improve glucose tolerance, decrease insulin resistance (HOMA-IR), and enhance GLUT4 translocation in skeletal muscle [130,131,132,133]. Histopathological analyses further demonstrate preservation of pancreatic β-cell integrity, increased insulin immunoreactivity, and reduced oxidative stress within islet tissue, supporting a dual mechanism encompassing both insulin secretagogue and insulin-sensitizing effects [134]. Collectively, these findings position basil as promising adjuncts in T2DM management, particularly in resource-constrained LMIC settings; although, larger, well-controlled clinical trials remain necessary to establish long-term efficacy and safety.

#### 3.3.5. Anticancer Activity

In studies conducted on cancer cell lines, basil species have exhibited anticancer activities over HeLa, Hep-2, HepG2, HPV and MCF-7 cell lines [29,33,135,136,137]. Linalool demonstrated dose-dependent cytotoxicity (IC50: 75–150 µg/mL) whilst displaying selective toxicity favoring neoplastic over normal cell lines at equivalent concentrations [29,33]. Rosmarinic acid (IC50: 45–90 µM) induced apoptosis via intrinsic (mitochondrial) and extrinsic pathways. Upregulation of pro-apoptotic proteins (Bax, Bak, Bad), downregulation of Bcl-2/Bcl-xL, sequential caspase-3/-8/-9 activation, and PARP cleavage were consistently observed [138,139,140].

Additional anti-neoplastic effects include arrest of the cell cycle at both the G0/G1 and G2/M phases, mediated by downregulation of cyclin D1/CDK4 and cyclin B1/CDC2 [29]. The leaf extract also inhibits the expression of matrix metalloproteinases (MMP-2 and MMP-9), upregulates E-cadherin, indicating reversal of epithelial–mesenchymal transition (EMT), and exerts anti-angiogenic activity through suppression of vascular endothelial growth factor (VEGF) [29,141,142]. In vivo studies further support these findings. In Ehrlich ascites carcinoma models, treatment resulted in a 45–60% reduction in tumor volume at doses of 100 mg/kg intraperitoneal injection of ethanol extract from aerial parts. Similarly, in DMBA-induced skin carcinogenesis models, it delayed tumor onset and reduced tumor multiplicity [29,143].

In vitro and in vivo cytotoxicity against established cancer cell lines and carcinoma models does not constitute evidence of anticancer efficacy in humans. None of the 18 studies included reported data with pharmacokinetic validation. The pharmacokinetic achievability of effective cytotoxic concentrations at tumor sites in vivo cannot be translated to humans. These findings are preliminary and hypothesis-generating.

#### 3.3.6. Cardioprotective Activity

Sixteen of the included studies have documented varied cardiovascular protective effects. Hypolipidemic activity included reductions in total cholesterol (15–30%), LDL-C (20–35%), and triglycerides (25–40%), alongside HDL-C elevation (10–20%), partially attributable to HMG-CoA reductase inhibition [125,144]. Antihypertensive effects were mediated via ACE inhibition (IC50: 0.8–2.5 mg/mL), calcium channel blocking activity, and endothelium-dependent vasodilation through nitric oxide (NO) pathway enhancement [145,146]. Reduction of 15–25 mmHg systolic pressure was observed in hypertensive animal models [118]. Anti-atherosclerotic properties included inhibition of LDL oxidation, reduction in aortic fatty streak formation, and decreased foam cell genesis [147].

Direct cardiac protection was evidenced by reduced infarct size in ischemia–reperfusion models and anti-arrhythmic effects [148,149]. In myocardial infarction models, 40 mg/kg of basil leaf ethanolic extract two times per day was shown to strongly protect myocardium against isoproterenol-induced infarction [150]. All drug comparisons are within study references from the original publications and should not be interpreted as evidence of clinical equivalence. No clinical RCTs address cardiovascular endpoints. Human pharmacokinetic data are absent. Risk-of-bias limitations in animal studies likely inflate reported effect magnitudes.

#### 3.3.7. Neuroprotective Activity

Acetylcholinesterase (AChE) and butyrylcholinesterase (BChE) inhibition (IC50: 0.5–2 mg/mL) has been documented, which is mechanistically relevant to Alzheimer’s disease management. Reduction in amyloid-β (Aβ) aggregation and protection against 6-OHDA and MPTP-induced neurotoxicity were additionally reported [151,152,153,154]. Neuroinflammation was attenuated via suppression of microglial activation and pro-inflammatory cytokine expression [151,155]. Behaviorally, basil extracts exhibited significant anxiolytic activity in the elevated plus maze, antidepressant effects in the forced swim test comparable to fluoxetine (100–200 mg/kg), and cognitive function improvement in the Morris water maze where basil EO (1% *V*/*V*) was used as an inhalation agent, suggesting GABAergic and serotonergic modulation [156,157]. Out of the 13 included studies, no clinical trials address neuroprotective activity. Drug comparisons with fluoxetine are within-study parallels only.

#### 3.3.8. Wound Healing and Dermatological Applications

Thirteen of the included studies have evaluated wound healing efficacy. Accelerated wound closure (30–50% faster) in excision models, increased tensile strength in incision models, and reduced healing time in burn wound models were consistently reported [158,159,160]. Basil preparations enhanced fibroblast proliferation and collagen synthesis (proliferative phase), stimulated VEGF-dependent angiogenesis, and promoted keratinocyte migration (re-epithelialization) [161,162]. Antimicrobial protection of the wound bed against common colonizers (*S. aureus*, *P. aeruginosa*) complemented these healing effects [163,164]. A 3% EO topical formulation produced 51 mm inhibition zones against *Cutibacterium acnes*, supporting utility in acne vulgaris management [165,166]. Wound healing evidence is entirely preclinical. No clinical wound-healing trials were identified. Formulation, concentration, and application method varied considerably across included studies.

#### 3.3.9. Larvicidal and Vector Control Activity

Larvicidal activity against mosquito vectors of malaria, dengue, and lymphatic filariasis has also been documented in 19 of the included studies, reflecting its importance in the management of vector-borne disease in tropical LMIC contexts [83,167]. Field trials conducted in Nigeria, and Tanzania, demonstrating that basil extracts achieved more than 90% larval mortality against *Anopheles* species with independent replication across settings. In Malawi it has been documented as the most effective mosquito repellent [50,168,169,170]. The principal larvicidal compounds were identified as linalool oxide, camphor, and α-terpineol [83]. Basil essential oils target multiple physiological pathways, acting simultaneously on ion channels, olfactory receptors, and acetylcholinesterase in mosquitoes, which may help slow the development of resistance [171]. These could serve as valuable tools within Integrated Vector Management (IVM), particularly for resource-constrained national vector control programs.

#### 3.3.10. Additional Pharmacological Activities

Further activities reported in subsets of studies include gastroprotection against ethanol- and NSAID-induced gastric ulceration via mucus secretion enhancement and prostaglandin E2 elevation at a dose of 100–200 mg/kg of basil whole plant extract [172,173]; hepatoprotection against CCl4, paracetamol, and alcohol-induced hepatotoxicity (normalization of serum ALT, AST, ALP, and restoration of hepatic histoarchitecture) after basil supplementation at a dose of 100 mg/kg orally [95,174]. Basil extracts (100 mg/kg) have also shown to have nephroprotection activity against aminoglycoside (gentamicin) and platinum (cisplatin) nephrotoxicity [175,176], and bidirectional immunomodulation, immunostimulant effects at low doses (enhanced macrophage phagocytosis, NK cell activity) and immunosuppressant effects at high doses [94,177]. Basil extracts have also shown to reduce stress and improve sleep in humans at a dose of 125 mg (hydroalcoholic extract derived from the leaves’ rich aerial parts) twice a day taken orally [20]. Each of these activities is based on a small number of studies (n = 2) and should be considered preliminary evidence requiring replication.

### 3.4. Toxicity and Safety Profile

Acute oral toxicity studies classify basil aqueous extract as “practically non-toxic” under WHO criteria (LD_50_ > 5000 mg/kg in rats) [178]. Essential oil LD_50_ values range from 1.5 to 3 g/kg, varying with chemotype [179,180]. Ninety-day repeated-dose studies established a No Observed Adverse Effect Level (NOAEL) of 1000 mg/kg/day for aqueous extract [181]. Safety cannot be generalized across all *Ocimum* species, chemotypes, extract types, doses, routes of administration, or human populations. Aqueous extracts, hydroalcoholic extracts, essential oils, and isolated compounds have markedly different toxicity profiles.

The primary safety concern is estragole, a phenylpropanoid with demonstrated genotoxic hepatocarcinogenicity in rodents at pharmacologically excessive doses of 2000 mg/kg [182]. The Joint FAO/WHO Expert Committee on Food Additives (JECFA) has concluded that human dietary exposure is not of concern, but medicinal-grade preparations from estragole-rich chemotypes warrant dose limitation and explicit labeling [183]. Estragole-containing preparations should be avoided during pregnancy given demonstrated genotoxicity in rodent models. Eugenol exerts dose-dependent antiplatelet activity, necessitating caution in patients receiving anticoagulant therapy [184]. Potential herb–drug interactions include eugenol with anticoagulants (additive antiplatelet effect); camphor with CNS depressants (additive sedation risk); and cytochrome P450 modulation by multiple EO constituents [183,185].

Available evidence does not support generalized safety claims for pregnant or lactating women [185,186]. Clinical use in these populations should be considered only under medical supervision with chemotype-authenticated preparations. No pediatric pharmacokinetic or safety data are available. Chronic exposure data beyond 90-day NOAEL studies are absent. Adulteration and quality variability in commercially available preparations pose additional safety risks requiring standardized quality-control frameworks.

### 3.5. Ethnobotanical Documentation in LMICs

#### 3.5.1. African Ethnomedicinal Systems

Sub-Saharan Africa, has extraordinary botanical diversity and the deep embeddedness of traditional healing practices across diverse cultural systems [187]. Across West Africa, O. *basilicum*, *O. gratissimum*, *O. americanum*, *O. tenuiflorum* are the most socioculturally prominent medicinal *Ocimum* species. Known as Nchanwu in Igbo-speaking communities of southeastern Nigeria and Efirin in Yoruba-speaking southwest Nigeria, its leaves are used as decoctions for the management of fever, diarrhea, skin infections, and infertility, a range of applications corroborated by pharmacological evidence for antimicrobial, antipyretic, and hormonal-modulatory activities [9,14,15,188].

In East and Southern Africa, basil has long been integrated into traditional medicine systems and is widely recognized by different local names across communities. In Malawi, it is commonly known as Mpungabwi; in Tanzania and Kenya, as Mrehani; and in Uganda, as Mujaaj. Basil species including *O. canum*, *O. kilimandscharicum* and *O. americanum* feature prominently in respiratory and parasitic disease management. Ethnobotanical surveys from these regions have documented that traditional healers and communities at large, primarily use steam inhalation of basil for chronic cough and asthma, and topical leaf ointment for ectoparasite elimination, applications pharmacologically consistent with the species’ camphor-dominant and 1,8-cineole-rich EO profile [9,10,26,27,188].

In north Africa particularly Egypt, basil has been used for centuries for both culinary and medicinal purposes, with archeological evidence indicating its cultural significance, particularly for *O. sanctum* and O. *basilicum*. Historically, basil is used medicinally for gastrointestinal disorders, respiratory ailments, wound care, and general therapeutic purposes within traditional healing systems [39].

#### 3.5.2. South and Southeast Asian Ethnomedicinal Systems

The Ayurvedic tradition of the Indian subcontinent represents the most codified and historically documented ethnomedicinal system engaging *Ocimum* species. *O. tenuiflorum* occupies a uniquely revered status within Hindu religious and therapeutic cosmology [13]. Basil has been used in Chinese medicine as an adaptogen, immunomodulator, and treatment for respiratory, metabolic, and neurological ailments [22,23]. This deep cultural integration facilitates high community acceptability and accessibility, characteristics that make it an especially promising candidate for LMIC primary healthcare formalization [189].

In Thailand and Cambodia, Southeast Asia nations with traditional medicine infrastructure, *O. tenuiflorum* and *O. basilicum* are both culinary staples and medicinal resources. Thai traditional practitioners document their use in management of type 2 diabetes, flatulence, nausea, dysmenorrhea, and as postnatal tonics [190,191,192].

## 4. Discussion

This systematic review consolidates a substantial and multidisciplinary body of evidence demonstrating that *Ocimum* species possess a diverse array of pharmacologically relevant biological activities with meaningful implications for primary healthcare delivery in low- and middle-income countries. Across the domains of antimicrobial, antioxidant, anti-inflammatory, antidiabetic, anticancer, cardioprotective, neuroprotective, wound healing, and vector control activity, evidence drawn from in vitro, in vivo, and selected clinical investigations consistently corroborates the ethnobotanical rationale supporting the widespread traditional use of these plants [9,10,25,44]. The convergence of ethnobotanical documentation with mechanistic preclinical pharmacology across sub-Saharan Africa, the Indian subcontinent, and Southeast Asia is among the most significant observations of this review. Traditional use patterns do not merely constitute historical interest, they represent a culturally validated hypothesis-generating framework whose pharmacological coherence has been repeatedly confirmed in vitro and in vivo [13,14,26,27]. However, with a predominantly preclinical evidence base, this review does not establish therapeutic efficacy in humans for any pharmacological domain, it establishes biological plausibility and identifies priority areas for future clinical investigation.

### 4.1. Priority Domains and Key Bioactive Compounds

Among the reviewed species, *Ocimum tenuiflorum* and *Ocimum gratissimum* emerge as leading candidates for further translational research due to their extensive pharmacological evidence base, widespread availability across high-burden LMIC settings, and relevance to prevailing infectious and metabolic health challenges (Table 3). *Ocimum canum* and *Ocimum kilimandscharicum* warrant prioritization for vector-control strategies, supported by promising field-based larvicidal efficacy. In contrast, *Ocimum basilicum* demonstrates particular potential for antioxidant and antimicrobial applications, especially in Southeast Asia and North Africa, where its cultivation and traditional use are well established [12,35,44,49,52,56].

The phytochemical architecture of basil is characterized by both breadth and chemotypic plasticity. The essential oil fraction, dominated by linalool, eugenol, estragole, camphor, 1,8-cineole, and methyl chavicol, varies markedly across species, geographic accessions, and developmental stages, a variability that confounds direct cross-study pharmacological comparisons and emphasizes the necessity of chemotype authentication in future research [57,62,63]. The non-volatile fraction, comprising rosmarinic acid, caffeic acid, apigenin, luteolin, quercetin, ursolic acid, and oleanolic acid, is of equal pharmacological relevance [86,88,91,93]. Rosmarinic acid in particular, consistently identified at concentrations of 2–15 mg/g dry weight across *O. basilicum*, *O. tenuiflorum*, and *O. americanum* accessions, demonstrates pleiotropic bioactivity spanning radical scavenging, COX-2 inhibition, NF-κB suppression, and pro-apoptotic induction [32,87,93,136]. Notably, tropical LMIC-derived germplasm exhibits significantly higher total phenolic content (48.2 mg GAE/g DW) than temperate cultivars (28.7 mg GAE/g DW), with correspondingly enhanced antioxidant capacity (DPPH IC_50_: 12.4 vs. 24.6 µg/mL) [111], an observation attributable to environmental upregulation of phenylpropanoid biosynthesis [112,113], and one that challenges the assumption that LMIC-derived germplasm exhibits inferior phytochemical quality relative to temperate cultivars. Key bioactive markers, rosmarinic acid, eugenol, linalool, ursolic acid, and β-caryophyllene, demonstrate significant bioactivity spanning across antimicrobial, antioxidant, anti-inflammatory, antidiabetic, and anticancer domains.

Antimicrobial findings carry particular epidemiological weight in LMIC contexts, where infectious diseases remain leading causes of morbidity and mortality. Broad-spectrum antibacterial activity has been consistently demonstrated in vitro, with minimum inhibitory concentrations (MICs) of 0.31–1.25 mg/mL for eugenol-rich *O. gratissimum* essential oil against Salmonella typhimurium, Vibrio cholerae, and Staphylococcus aureus [97,98]. Importantly, methicillin-resistant Staphylococcus aureus (MRSA) was consistently susceptible (MIC: 0.6–2.5 mg/mL), with eugenol and linalool identified as mediators of membrane disruption and biofilm inhibition [102,103,104], findings of direct relevance given escalating global antimicrobial resistance. Antifungal efficacy against systemic candidiasis, Aspergillus spp., and dermatophytes further broadens the therapeutic scope [99,106]. Nevertheless, substantial inter-study heterogeneity in MIC values, driven by variability in chemotype, extraction method, and assay format [100,101], and the near-universal reliance on in vitro models remain critical limitations constraining clinical translation.

Antioxidant capacity is among the most technically robust findings, with DPPH IC_50_ values of 12.5–89.3 µg/mL attributed principally to rosmarinic acid and phenolic acid constituents [32,33,108,109,110]. A strong positive correlation between total phenolic content and radical-scavenging activity (r^2^ = 0.89, *p* < 0.001) [32,114,115], coupled with in vivo evidence of enhanced superoxide dismutase, catalase, and glutathione peroxidase activity [116,117,118], validates the translational plausibility of these findings. Anti-inflammatory activity is mechanistically anchored in NF-κB p65 suppression and concurrent Nrf2/HO-1 pathway activation [14,37], with eugenol, ursolic acid, and β-caryophyllene identified as the principal bioactive mediators. In vivo reductions in carrageenan-induced paw edema of 35–55% [19,24] are of direct relevance to the infectious and non-communicable inflammatory disease burden characteristic of LMIC populations.

Antidiabetic activity warrants particular strategic emphasis, given that approximately 80% of the global type 2 diabetes mellitus burden is concentrated in LMICs [119]. Inhibition of α-glucosidase (IC_50_: 0.3–1.5 mg/mL) and α-amylase (IC_50_: 0.5–2.0 mg/mL) comparable to acarbose [122,123,124], combined with in vivo fasting blood glucose reductions of 25–40% and preservation of pancreatic β-cell integrity in streptozotocin-induced models [126,127,128,129,130], provides a coherent mechanistic basis for the traditional management of diabetes using *Ocimum* preparations. Additional pharmacological domains, including cardio protection through ACE inhibition and lipid-lowering effects [121,140,141,142], neuroprotection via acetylcholinesterase inhibition and amyloid-β reduction [146,147,148,149], wound healing acceleration through enhanced fibroblast proliferation and VEGF-dependent angiogenesis [153,154,155,156,157], and pro-apoptotic anticancer activity across multiple cell lines [29,134,135,136], collectively reinforce the therapeutic versatility of the genus, though each requires further validation in clinically relevant models.

The larvicidal evidence warrants particular attention within the LMIC vector control context. Field trials conducted in Nigeria and Tanzania demonstrated greater than 90% larval mortality against *Anopheles* spp. [163,164,165], with linalool oxide, camphor, and α-terpineol identified as principal larvicidal compounds [79], and *O. canum* documented as the most effective repellent in Malawi [50]. The multi-target physiological mode of action of basil EOs on mosquito vectors [166] confers resistance-management advantages relevant to national Integrated Vector Management (IVM) programs. The safety profile of the genus is broadly favorable (acute oral LD_50_ > 5000 mg/kg; NOAEL 1000 mg/kg/day) [173,176], with caution, estragole-related genotoxicity in high-estragole chemotypes, eugenol-mediated antiplatelet activity, absence of reproductive and pediatric safety data, and potential herb–drug interactions require management through chemotype-aware standardization [177,178,179].

Larvicidal field evidence is the most directly translatable to LMIC practice of all pharmacological domains reviewed, with consistent effect sizes across independent field settings. Key remaining gaps include formulation optimization, environmental persistence, and safety to non-target organisms.

### 4.2. Translation Barriers and Transition Roadmap

Several key translation barriers must be addressed before *Ocimum*-derived interventions can be advanced into clinical practice. First, substantial chemotypic variability and the lack of extract standardization contribute to considerable inter-study heterogeneity, limiting reproducibility, comparability, and product development. Second, critical pharmacokinetic parameters, including oral bioavailability, first-pass metabolism, and tissue distribution of major bioactive constituents, remain poorly characterized in humans. Third, despite promising preclinical findings, adequately powered randomized controlled trials evaluating priority disease indications in LMIC populations are lacking. Finally, regulatory frameworks governing plant-based medicines remain underdeveloped in many LMIC settings, creating additional barriers to clinical translation and implementation.

**Figure 4:**
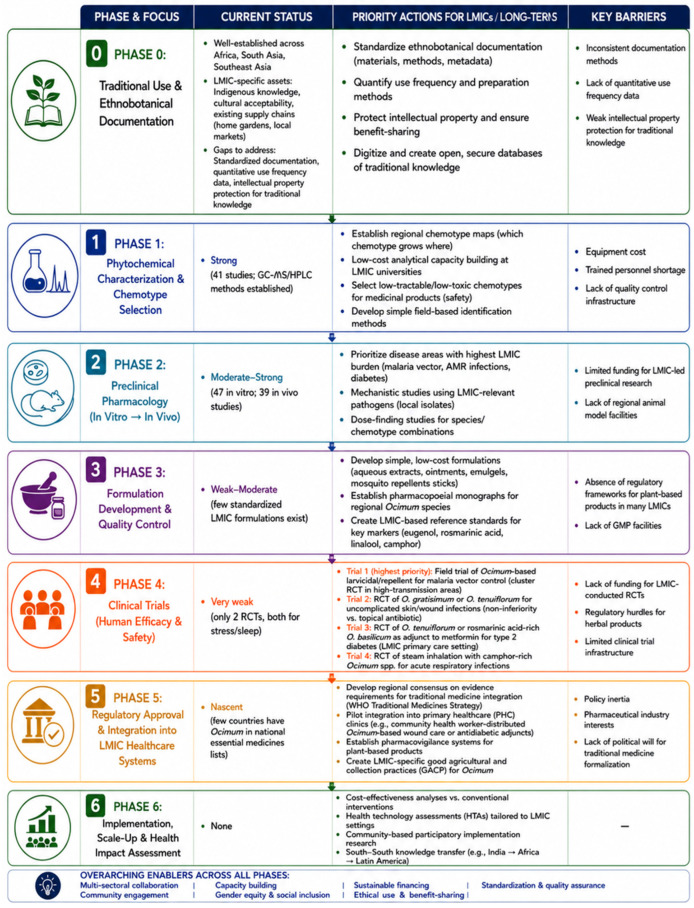
Roadmap for the translation of *Ocimum* Species from traditional use to healthcare integration in LMICs. **Note:** AMR: antimicrobial resistance; GACP: good agricultural and collection practices; GC–MS: gas chromatography–mass spectrometry; GMP: good manufacturing practice; HPLC: high-performance liquid chromatography; HTA: health technology assessment; LMICs: low- and middle-income countries; PHC: primary healthcare; RCT: randomized controlled trial; WHO: World Health Organization.

To address these challenges, a phased translational pathway is proposed. The first stage should focus on chemotype authentication and standardized extract characterization. This should be followed by pharmacokinetic and bioavailability studies to establish exposure profiles of key bioactive compounds. The third stage should involve comprehensive safety assessment, including evaluation of herb–drug interactions and reproductive toxicity. Subsequently, LMIC-contextualized Phase II RCTs should be undertaken for priority disease species. The final stage should encompass cost-effectiveness, implementation, and health systems research to support sustainable integration into routine healthcare delivery (Figure 4).

The roadmap emphasizes LMIC-specific opportunities, including the use of indigenous knowledge, locally relevant disease priorities, affordable formulation strategies, regional regulatory harmonization, and community-based implementation approaches. Overarching enablers across all phases include multi-sectoral collaboration, capacity building, sustainable financing, standardization and quality assurance, community engagement, gender equity and social inclusion, and ethical benefit-sharing. The framework identifies critical evidence gaps and provides a strategic pathway for translating *Ocimum*-based interventions into safe, effective, and accessible healthcare solutions in LMIC settings.

### 4.3. Risk-of-Bias Implications for Evidence Certainty

The risk-of-bias profile varied across evidence streams. Antimicrobial in vitro studies were generally of moderate-to-high methodological quality and yielded reproducible findings under controlled conditions. However, the absence of pharmacokinetic validation limits clinical extrapolation. In contrast, anti-inflammatory and antidiabetic animal studies were frequently affected by inadequate randomization, allocation concealment, and blinding, increasing the likelihood of inflated treatment effects. Consequently, reported effect sizes should be interpreted cautiously. Clinical evidence remains sparse and heterogeneous, with only two RCTs conducted in high-income settings, limiting applicability to LMIC populations. Larvicidal field studies provided the most robust translational evidence, demonstrating consistently high efficacy across independent settings. Overall, publication bias favoring positive natural-product findings likely inflates the apparent magnitude of effects across the evidence base.

### 4.4. Limitations

Despite the strong ethnobotanical relevance and pharmacological promise of *Ocimum* species in LMICs, several limitations constrain the translational strength of this review. Most included studies were in vitro or rodent-based, limiting direct extrapolation to human clinical settings. Considerable heterogeneity in chemotypes, extraction methods, solvent systems, phytochemical characterization, and assay conditions reduced inter-study comparability, while publication bias toward positive findings may have inflated reported efficacy. The search was restricted to English-language publications, introducing potential language bias that may underrepresent LMIC-language literature. Additionally, ethnobotanical evidence was largely derived from cross-sectional surveys prone to recall bias. The scarcity of adequately powered, LMIC-contextualized randomized clinical trials remains a critical evidence gap requiring standardized reporting and prospective methodological frameworks. Several pharmacological effects are supported primarily by preclinical evidence, with only two clinical studies. Therefore, further clinical validation and independent replication are required before robust conclusions can be drawn.

## 5. Conclusions

This systematic review establishes that *Ocimum* species constitute a pharmacologically diverse and preclinically well-supported botanical resource with genuine potential relevance for primary healthcare in LMICs. Basil extracts have consistently demonstrated antimicrobial, antioxidant, anti-inflammatory, and antidiabetic activities, while cardioprotective, neuroprotective, wound healing, anticancer, and larvicidal effects are corroborated by multiple independent investigations. The strong agreement between ethnobotanical documentation and experimental pharmacological findings across African, South Asian, and Southeast Asian healing traditions provides a scientifically defensible basis for exploring the formalization of evidence-validated *Ocimum*-based interventions within LMIC primary healthcare frameworks.

The evidence base does not, at present, support recommendations for clinical implementation. The evidence establishes biological plausibility and preclinical promise; it does not establish therapeutic efficacy, optimal dose, safety in diverse human populations, pharmacokinetics, drug interactions, or cost-effectiveness. The critical translational gap from in vitro characterization to adequately powered human clinical trials remain the foremost barrier to policy and practice integration. Toxicological concerns associated with estragole-rich chemotypes, and eugenol-mediated antiplatelet, absence of reproductive and pediatric safety data, and potential herb–drug interactions are manageable through chemotype-aware standardization but must not be understated. Future research must prioritize LMIC-contextualized randomized controlled trials, standardized phytochemical reporting, pharmacokinetic characterization in relevant populations, systematic safety reporting inclusive of herb–drug interactions, and phytochemical reporting aligned with established standards. When pursued with scientific rigor and equity-centered intent, *Ocimum* species hold meaningful promise as affordable, locally derived therapeutic resources for populations bearing the world’s most inequitably distributed disease burden.

## Figures and Tables

**Table 1:** Medicinally significant *Ocimum* species in LMICs.

Species	Primary Geographic Distribution (LMIC-Relevant)	Common Names	Key Traditional Uses	References
***Ocimum*** ***basilicum* L.**	Widespread; West and East Africa, South and Southeast Asia, parts of South America.	Sweet basil	Tea or decoction for common cold, cough, digestive disorders (diarrhea, dysentery, indigestion), hypertension, headache, rheumatism, and anti-helminthic use.	[12,44,45]
***Ocimum*** ***tenuiflorum***	South Asia, widely naturalized in tropical Africa, ASEAN, Oceania.	Holy basil, Tulsi, “Sacred basil”.	Adaptogen for stress, fever, respiratory infections (cough, bronchitis), malaria fever, headache, digestive disorders, arthritis, and venom-bite antidote.	[44,46,47]
***Ocimum*** ***gratissimum* L.**	Tropical Africa, West and Central Africa, India, South America and parts of Southeast Asia.	African basil, Clove basil, “Scent leaf” (West Africa).	Antidiabetic, antiseptic, antimicrobial, antidiarrheal, antipyretic; used for respiratory infections, fever, stomach/kidney ailments, skin infections, and convulsions/epilepsy in some areas.	[46,48,49]
***Ocimum*** ***americanum* L.**	Tropical Africa, India, Thailand, Malaysia, and West Africa.	Camphor basil, African camphor basil, “Hoary basil”	Respiratory ailments (cough, bronchial catarrh), hypertension, diabetes support, stomach pain, diarrhea, dysentery, hemorrhoids, and eye/ear complaints.	[44,46]
***Ocimum canum* Sims**	Widespread in tropical Africa, including Malawi, Nigeria, Ethiopia, India and Brazil	Malawi camphor basil, African camphor basil, “Camphor basil”.	Antidiabetic, antimicrobial, mosquito-repellent, antipyretic, colds, and respiratory-related complaints.	[50,51,52]
***Ocimum*** ***kilimandscharicum***	Eastern Africa (Tanzania, Kenya, Uganda), often grown in home gardens.	Tanzanian camphor basil, Kilimanjaro camphor basil. African Blue basil	Camphor-rich essential oil used for antifungal activity, mosquito repellent, respiratory-related decoctions, and topical antiseptic, mild adaptogenic/antimicrobial use in home-remedy teas; often used as ornamental and for aromatic properties.	[17,53,54,55,56]

Note: LMICs: low-and middle-income countries; ASEAN: Association of Southeast Asian Nations.

**Table 3:** Priority ranking of the *Ocimum* species of LMICs importance and key bioactive compounds.

Priority Rank	Species	Dominant Chemotype(s)	Key Bioactive Marker Compounds	Analytical Method for Standardization	Primary Traditional Use (LMIC)	Best Evidence Domain	Region/Country
**1**	*O. gratissimum* L.	Eugenol-rich (60–85%)	Eugenol, thymol, γ-terpinene, p-cymene	GC-MS (EO); HPLC-UV (phenolics)	Malaria, wound infections, diarrhea, diabetes	Antimicrobial, antidiabetic	Tropical Africa, Brazil, India
**2**	*O. tenuiflorum* L.	Eugenol-rich (up to 71%) OR methyl eugenol-rich	Eugenol, β-caryophyllene, ursolic acid, rosmarinic acid, orientin, vicenin-2	GC-MS (EO); HPLC-DAD (phenolics, flavonoids)	Stress, diabetes, respiratory infections, fever	Anti-inflammatory, antidiabetic, adaptogen	South Asia, Southeast Asia
**3**	*O. basilicum* L.	Linalool-rich (35–65%) OR estragole-rich (30–45%)	Linalool, rosmarinic acid, estragole, quercetin, apigenin	GC-MS (EO); HPLC-DAD (phenolics)	Digestive disorders, cough, headache, fever	Antimicrobial, antioxidant, cardioprotective	Global
**4**	*O. canum* Sims	Camphor + carvacrol + p-cymene	Carvacrol, camphor, p-cymene, γ-terpinene	GC-MS	Mosquito repellent, antidiabetic, respiratory	Larvicidal/vector control, antidiabetic	East/Southern Africa
**5**	*O. americanum* L.	1,8-Cineole-rich (51.8%) + camphor (17.4%)	1,8-Cineole, camphor, linalool	GC-MS	Respiratory ailments, hypertension, diabetes	Antimicrobial, respiratory relief	Tropical Africa, Southeast Asia
**6**	*O. kilimandscharicum*	Camphor-rich (40–65%) + 1,8-cineole (15–30%)	Camphor, 1,8-cineole, α-thujene	GC-MS	Respiratory conditions, mosquito repellent, antiseptic	Antifungal, larvicidal	East Africa

**Note:** DAD: diode array detection; EO: essential oil; GC-MS: gas chromatography–mass spectrometry; HPLC: high-performance liquid chromatography; LMIC: low- and middle-income country; UV: ultraviolet.

## Data Availability

No new data were created or analyzed in this study. Data sharing is not applicable to this article.

## Supplementary Materials

The following supporting information can be downloaded at: https://www.mdpi.com/article/10.3390/ijms27125540/s1.

## Abbreviations

The following abbreviations are used in this manuscript:

**6-OHDA**	6-Hydroxydopamine
**ABTS**	2,2-azino-bis-3-ethylbenzothiazoline-6-sulfonic acid
**ACE**	Angiotensin-Converting Enzyme
**AChE**	Acetylcholinesterase
**ALP**	Alkaline Phosphatase
**ALT**	Alanine Aminotransferase
**AST**	Aspartate Aminotransferase
**Aβ**	Amyloid-beta
**BChE**	Butyrylcholinesterase
**CAT**	Catalase
**CCl4**	Carbon Tetrachloride
**CDC2**	Cell Division Cycle 2 kinase
**CDK4**	Cyclin-Dependent Kinase 4
**COX-1**	Cyclooxygenase-1
**COX-2**	Cyclooxygenase-2
**CUPRAC**	Cupric Reducing Antioxidant Capacity
**DMBA**	7,12-Dimethylbenz[a]anthracene
**DPP-IV**	Dipeptidyl Peptidase-IV
**DPPH**	2,2-diphenyl-1-picrylhydrazyl
**DW**	Dry Weight
**EMT**	Epithelial–Mesenchymal Transition
**EO**	Essential Oil
**FRAP**	Ferric Reducing Antioxidant Power
**GABAergic**	Gamma-Aminobutyric Acid-ergic (relating to GABA neurotransmission)
**GAE**	Gallic Acid Equivalents
**GC-MS**	Gas Chromatography–Mass Spectrometry
**GLUT4**	Glucose Transporter Type 4
**GPx**	Glutathione Peroxidase
**GR**	Glutathione Reductase
**HDL-C**	High-Density Lipoprotein Cholesterol
**HMG-CoA**	3-Hydroxy-3-Methylglutaryl Coenzyme A
**HO-1**	Heme Oxygenase-1
**HOMA-IR**	Homeostatic Model Assessment of Insulin Resistance
**HPLC-DAD**	High-Performance Liquid Chromatography with Diode Array Detection
**HPV**	Human Papillomavirus
**IC_50_**	Half Maximal Inhibitory Concentration
**IL-1α**	Interleukin-1 beta
**IL-6**	Interleukin-6
**IVM**	Integrated Vector Management
**LC-MS**	Liquid Chromatography-Mass Spectrometry
**LD_50_**	Median Lethal Dose
**LDL-C**	Low-Density Lipoprotein Cholesterol
**LMICs**	Low- and Middle-Income Countries
**LOX**	Lipoxygenase
**LPS**	Lipopolysaccharide
**MDA**	Malondialdehyde
**MeSH**	Medical Subject Headings
**MIC**	Minimum Inhibitory Concentration
**MMP-2**	Matrix Metalloproteinase-2
**MMP-9**	Matrix Metalloproteinase-9
**MPTP**	1-Methyl-4-Phenyl-1,2,3,6-Tetrahydropyridine
**MRSA**	Methicillin-Resistant Staphylococcus aureus
**NF-kB**	Nuclear Factor kappa-light-chain-enhancer of activated B cells
**NK**	Natural Killer (cells)
**NO**	Nitric Oxide
**Nrf2**	Nuclear factor erythroid 2-related factor 2
**NSAID(s)**	Nonsteroidal Anti-Inflammatory Drug(s)
**PARP**	Poly ADP-ribose Polymerase
**SOD**	Superoxide Dismutase
**T2DM**	Type 2 Diabetes Mellitus
**TBARS**	Thiobarbituric Acid-Reactive Substances
**TNF-alpha**	Tumor Necrosis Factor-alpha
**TPC**	Total Phenolic Content
**TRPV1**	Transient Receptor Potential Vanilloid 1
**VEGF**	Vascular Endothelial Growth Factor
