# Supplementary material for: Ethnobotany, Phytochemistry, and Pharmacological Activities of Ocimum Species in Low- and Middle-Income Countries: A Systematic Review
Source: Int J Mol Sci. 2026 Jun 18;27(12):5540. doi: 10.3390/ijms27125540 (PMC13300166; doi:10.3390/ijms27125540)
Supplement: Supplementary file 1 [file ijms-27-05540-s001.zip › ijms-4354161-supplementary.pdf]

Supplementary Table S1. Complete Boolean search strings of each database.

Database	Search String	Filters / Limits	Results Retrieved
**PubMed**	(*"Ocimum"* OR *"Ocimum basilicum"* OR *"Ocimum tenuiflorum"* OR *"Ocimum gratissimum" OR "Ocimum americanum"* OR *"Ocimum canum"* OR *"Ocimum kilimandscharicum"* OR "basil" OR "holy basil" OR "tulsi") AND ("pharmacology" OR "pharmacological" OR "bioactivity" OR "therapeutic" OR "antioxidant" OR "antimicrobial" OR "anti-inflammatory" OR "anticancer" OR "antidiabetic" OR "neuroprotective" OR "cardioprotective") AND ("extract" OR "essential oil" OR "phytochemical" OR "compound" OR "constituent")	Publication date: 2010/01/01 – 2025/12/31; English language; Humans or Animals	847
**Scopus**	("*Ocimum basilicum*" OR "*Ocimum tenuiflorum*" OR *"Ocimum gratissimum"* OR "*Ocimum americanum*" OR "*Ocimum canum*" OR *"Ocimum kilimandscharicum"* OR "basil" OR "holy basil" OR tulsi) AND ("pharmacology" OR "pharmacological" OR "bioactivity" OR "therapeutic" OR "antioxidant" OR "antimicrobial" OR "anti-inflammatory" OR "anticancer" OR "antidiabetic" OR "neuroprotective" OR "cardioprotective") AND ("extract" OR "essential oil" OR "phytochemical" OR "compound" OR "constituent")	PUBYEAR > 2009 AND PUBYEAR < 2026; Language: English; Document type: Article	1203
**Embase**	(*ocimum*/exp OR *ocimum basilicum* OR *ocimum tenuiflorum* OR *ocimum gratissimum* OR *ocimum americanum* OR *ocimum canum* OR *ocimum kilimandscharicum* OR basil OR holy basil OR tulsi) AND (pharmacology/exp OR pharmacological OR bioactivity OR therapeutic OR antioxidant OR antimicrobial OR anti-inflammatory OR anticancer OR antidiabetic OR neuroprotective OR cardioprotective) AND (extract OR essential oil OR phytochemical OR compound OR constituent)	Publication year: 2010-2025; Language: English; Article type: Original research	987

Database	Search String	Filters / Limits	Results Retrieved
**Web of Science**	(“*Ocimum basilicum*” OR “*Ocimum tenuiflorum*” OR “*Ocimum gratissimum*” OR “*Ocimum americanum*” OR “*Ocimum canum*” OR “*Ocimum kilimandscharicum*” OR “basil” OR “holy basil” OR “tulsi”) AND (“pharmacology” OR “pharmacological” OR “bioactivity” OR “therapeutic” OR “antioxidant” OR “antimicrobial” OR "anti-inflammatory" OR “anticancer” OR “antidiabetic” OR “neuroprotective” OR “cardioprotective”) AND (extract OR "essential oil" OR phytochemical OR compound OR constituent)	Publication years: 2010-2025; Language: English; Document type: Article	631
**Google Scholar**	Ocimum OR basil AND (pharmacology OR antimicrobial OR antioxidant OR anti-inflammatory OR antidiabetic OR anticancer OR therapeutic OR neuroprotective OR cardioprotective) AND (extract OR essential oil OR phytochemical OR compound OR constituent)	Since 2010; First 300 records screened (30 pages at 10 results per page); Clean browser session (cookies cleared, no login, fixed institutional IP); Single search date: 22 March 2026	5 (supplementary)

Supplementary Table S2. Detailed Characteristics of Included Studies (n=97) with Country, Income Classification, Plant Material, Study Design, Key Quantitative Outcome, and Methodological Quality Indicators.

Author	**Country of Research**	**World Bank Income Classification (2024-2025)**	**Species / Preparation**	**Plant Material Used**	**Study Design**	**Organisms / Model / Cell Line**	**Key Quantitative Outcome**	**Methodological Quality Indicators[1]**
**Alves-Silva et al. (2013) [101]**	Portugal	High income	*O. basilicum* EO	Aerial parts, hydrodistillation	In vitro	*S. aureus, S. typhimurium,* others	MIC: 0.31–1.25 mg/mL	Modified CONSORT: 6/8 (blinding NR, sample size justification NR)
**Elzaiat et al. (2024) [102]**	Egypt	Lower-middle income	Basil cultivar extracts	Leaves, solvent extraction	In vitro	Bacteria & fungi	MIC/inhibition zones quantified	Modified CONSORT: 5/8 (replicates unclear, blinding NR)
**Ajmal et al. (2025) [103]**	Pakistan	Lower-middle income	Basil EOs	Aerial parts, hydrodistillation	In vitro	*Aspergillus flavus, Fusarium moniliforme*	Antifungal & anti-aflatoxigenic MICs	Modified CONSORT: 7/8 (blinding NR)
**Sultan et al. (2024) [104]**	Iraq	Lower-middle income	*O. basilicum* organic extract & EO	Leaves, solvent extraction & hydrodistillation	In vitro	Mixed bacteria	MIC range: 0.3–5 mg/mL	Modified CONSORT: 5/8 (controls unclear, blinding NR)
**Sandulachi et al. (2023) [105]**	Moldova	Upper-middle income	Basil, thyme, tarragon extracts	Leaves, solvent extraction	In vitro	Food-associated pathogens	Inhibition data reported	Modified CONSORT: 4/8 (quantitative detail limited)
**Long et al. (2025) [106]**	China	Upper-middle income	Linalool (isolated compound)	Isolated from Ocimum	In vitro	MRSA	MIC: 0.6–2.5 mg/mL; biofilm inhibition	Modified CONSORT: 8/8 (full reporting)
**Wen et al. (2026) [107]**	China	Upper-middle income	Linalool + tetracycline	Isolated compound + standard drug	In vitro	MDR *S. aureus*	Synergistic MIC; biofilm disruption	Modified CONSORT: 7/8 (blinding NR)

Author	**Country of Research**	**World Bank Income Classification (2024-2025)**	**Species / Preparation**	**Plant Material Used**	**Study Design**	**Organisms / Model / Cell Line**	**Key Quantitative Outcome**	**Methodological Quality Indicators¹**
**El-Far et al. (2021) [108]**	Egypt	Lower-middle income	Eugenol (isolated compound)	Isolated from Ocimum	In vitro	MRSA clinical isolates (Egypt)	Biofilm inhibition & gene expression	Modified CONSORT: 7/8
**Amin et al. (2023) [109]**	Iran	Upper-middle income	Herbal EOs including Ocimum	Aerial parts, hydrodistillation	In vitro	Gram+ and Gram- bacteria	E. coli MIC: 1.5–4 mg/mL; P. aeruginosa MIC: 2–6 mg/mL	Modified CONSORT: 6/8
**Gupta et al. (2026) [110]**	India	Lower-middle income	*O. basilicum* & *O. sanctum* extracts	Leaves, solvent extraction	In vitro + In vivo	Candida spp.; systemic candidiasis (mouse)	MIC: 0.5–2 mg/mL; in vivo efficacy	In vitro: 6/8; SYRCLE: moderate RoB
**Akintunde et al. (2025) [78]**	Nigeria	Lower-middle income	*O. gratissimum* aqueous leaf extract	Leaves, aqueous extraction	In vitro	*S. aureus, P. aeruginosa*	Inhibition zones quantified	Modified CONSORT: 5/8
**Hao (2024) [75]**	Vietnam	Lower-middle income	*O. gratissimum* EO	Aerial parts, hydrodistillation	In vitro	Multiple bacteria	MIC values reported	Modified CONSORT: 5/8
**Chaturvedi et al. (2018) [54]**	India	Lower-middle income	*O. kilimandscharicum* EO	Aerial parts, hydrodistillation	In vitro	Bacteria & fungi	MIC + antioxidant IC50 reported	Modified CONSORT: 7/8
**Ali et al. (2022) [85]**	Kenya	Lower-middle income	*O. americanum* extracts	Leaves, solvent extraction	In vitro	Bacteria, fungi	MIC + cytotoxicity IC50	Modified CONSORT: 6/8
**da Silva et al. (2018) [52]**	Brazil	Upper-middle income	*O. canum* EO	Aerial parts, hydrodistillation	In vitro	*Leishmania amazonensis* + bacteria	Antimicrobial/antiprotozoal quantitative data	Modified CONSORT: 7/8
**Mahendran & Vimolmangkang (2023) [83]**	Thailand	Upper-middle income	*O. americanum* & *O. basilicum* EOs	Leaves, hydrodistillation	In vitro	Bacteria + mosquito larvae	MIC + LC50 for larvicidal	Modified CONSORT: 7/8

Author	Country of Research	World Bank Income Classification (2024-2025)	Species / Preparation	Plant Material Used	Study Design	Organisms / Model / Cell Line	Key Quantitative Outcome	Methodological Quality Indicators[1]
**Mohamed Abdoul-Latif et al. (2022) [84]**	Djibouti	Lower-middle income	*O. basilicum* & *O. americanum* EOs	Aerial parts, hydrodistillation	In vitro	Bacteria + cytotoxicity assays	MIC + cytotoxicity IC50	Modified CONSORT: 6/8
**Buragohain et al. (2026) [73]**	India	Lower-middle income	*O. tenuiflorum* EO	Aerial parts, hydrodistillation	In vitro	MDR *Aeromonas* spp.	MIC values reported	Modified CONSORT: 6/8
**Bsharat et al. (2026) [70]**	Jordan	Upper-middle income	Three basil cultivar EOs	Leaves, hydrodistillation	In vitro	Bacteria & fungi	MIC + antioxidant IC50	Modified CONSORT: 7/8
**Dharsono et al. (2022) [87]**	Indonesia	Upper-middle income	Multiple *Ocimum* spp. compounds	Various plant parts	In vitro	Bacteria	Comprehensive MIC data	Modified CONSORT: 6/8
**Poulios et al. (2025) [33]**	Greece	High income	*O. basilicum* extracts	Leaves, solvent extraction	In vitro	Multiple pathogens	MIC + antioxidant + anticancer IC50	Modified CONSORT: 8/8
**Ouandaogo et al. (2024) [18]**	Kenya/Burkina Faso	Lower-middle/Low income	Silver NPs from *O. kilimandscharicum*	Leaves, nanoparticle synthesis	In vitro	Selected bacteria	MIC reported	Modified CONSORT: 6/8
**Nadeem et al. (2022) [32]**	Pakistan	Lower-middle income	*O. basilicum* leaf extracts	Leaves, solvent extraction	In vitro	DPPH, ABTS, TPC assays	DPPH IC50: 15.2–67.8 μg/mL; r^2 = 0.89	Modified CONSORT: 7/8
**Poulios et al. (2025) [33]**	Greece	High income	*O. basilicum* extracts	Leaves, solvent extraction	In vitro	DPPH + antimicrobial + anticancer	IC50 range: 12.5–89.3 μg/mL	Modified CONSORT: 8/8
**Wojciak et al. (2024) [97]**	Poland	High income	*O. basilicum* polyphenolic fraction	Leaves, fractionation	In vitro	DPPH, ABTS, COX inhibition	Polyphenols: 60–70% of DPPH activity; COX IC50 quantified	Modified CONSORT: 8/8

Author	**Country of Research**	**World Bank Income Classification (2024-2025)**	**Species / Preparation**	**Plant Material Used**	**Study Design**	**Organisms / Model / Cell Line**	**Key Quantitative Outcome**	**Methodological Quality Indicators[1]**
**Coulibaly et al. (2023) [74]**	Burkina Faso	Low income	*O. gratissimum* EO	Aerial parts, hydrodistillation	In vitro	DPPH assay	IC50 values reported	Modified CONSORT: 5/8
**Chaturvedi et al. (2018) [54]**	India	Lower-middle income	*O. kilimandscharicum* EO	Aerial parts, hydrodistillation	In vitro	Antioxidant + antibacterial	IC50 values quantified	Modified CONSORT: 7/8
**Anusmitha et al. (2022) [88]**	India	Lower-middle income	Multiple *Ocimum* spp. extracts	Leaves, ultrasound-assisted extraction	In vitro	DPPH, anti-inflammatory, anticancer	IC50 for multiple activities	Modified CONSORT: 7/8
**Chaudhary et al. (2020) [112]**	India	Lower-middle income	*O. sanctum* extracts	Leaves, solvent extraction	In vitro	DPPH, TPC	IC50 and TPC values reported	Modified CONSORT: 6/8
**Abdel-Razakh et al. (2024) [114]**	Chad	Low income	*O. basilicum*	Leaves, solvent extraction	In vitro	DPPH, TPC, TFC	Phenolic content and IC50 values	Modified CONSORT: 5/8
**Tadevosyan et al. (2024) [28]**	Armenia	Upper-middle income	Multiple basil varieties	Leaves, solvent extraction	In vitro	TPC, TFC, antioxidant assays	Comparative IC50 values	Modified CONSORT: 6/8
**Yibeltal et al. (2022) [69]**	Ethiopia	Low income	Ethiopian *O. basilicum* EO & extract	Leaf & flower, hydrodistillation + solvent	In vitro	DPPH, antimicrobial	IC50 and MIC reported	Modified CONSORT: 6/8
**Qamar et al. (2023) [118]**	Pakistan	Lower-middle income	*O. basilicum* extract	Leaves, solvent extraction	In vitro + In vivo	DPPH + L-NAME hypertensive rats	DPPH IC50; SBP reduction 15–25 mmHg	In vitro: 7/8; SYRCLE: moderate RoB
**Othman et al.**	Saudi Arabia	High income	*O. basilicum* flavonoid fraction	Leaves, fractionation	In vivo	Diabetic albino rats	SOD, CAT, GPx enhancement; MDA reduction	SYRCLE: low-moderate RoB

Author	Country of Research	World Bank Income Classification (2024-2025)	Species / Preparation	Plant Material Used	Study Design	Organisms / Model / Cell Line	Key Quantitative Outcome	Methodological Quality Indicators¹
**(2021) [120]**								
**Teofilovic et al. (2021) [121]**	Serbia	Upper-middle income	*O. basilicum* extract	Aerial parts, solvent extraction	In vivo	Acetaminophen hepatotoxicity (rats)	ALT, AST normalization; antioxidant enzyme recovery	SYRCLE: moderate RoB
**Ben Mansour et al. (2024) [122]**	Tunisia	Lower-middle income	*O. basilicum* seed extract	Seeds, solvent extraction	In vivo	CCl4 renal toxicity (rats)	Serum creatinine, MDA, antioxidant enzyme values	SYRCLE: moderate RoB
**Wojciak et al. (2024) [97]**	Poland	High income	*O. basilicum* polyphenolic fraction	Leaves, fractionation	In vitro	LPS-stimulated RAW 264.7 macrophages	COX-1/COX-2 IC50: 25–80 μg/mL; LOX IC50: 15–60 μg/mL	Modified CONSORT: 8/8
**Anusmitha et al. (2022) [88]**	India	Lower-middle income	Multiple *Ocimum* spp.	Leaves, ultrasound-assisted extraction	In vitro	Multiple macrophage models	IC50 for anti-inflammatory activity	Modified CONSORT: 7/8
**Kamelnia et al. (2023) [94]**	Iran	Upper-middle income	*O. basilicum* & constituents	Various extracts & isolated compounds	In vitro	Macrophage models	Quantitative suppression of TNF-α, IL-1β, IL-6	Modified CONSORT: 7/8
**Ahmad & Iqbal (2025) [19]**	Pakistan	Lower-middle income	*Ocimum* spp. (100–200 mg/kg)	Leaves, solvent extraction	In vivo	Carrageenan paw oedema; cotton pellet granuloma	Oedema reduction 35–55%; granuloma reduction 35–50%	SYRCLE: moderate RoB

Author	Country of Research	World Bank Income Classification (2024-2025)	Species / Preparation	Plant Material Used	Study Design	Organisms / Model / Cell Line	Key Quantitative Outcome	Methodological Quality Indicators[1]
**El-Beshbishy & Bahashwan (2012) [128]**	Saudi Arabia	High income	*O. basilicum* aqueous extract	Leaves, aqueous extraction	In vitro	α-glucosidase & α-amylase inhibition	α-Glucosidase IC50: 0.3–1.5 mg/mL; α-amylase IC50: 0.5–2 mg/mL	Modified CONSORT: 6/8
**Wang et al. (2022) [126]**	USA	High income	*Ocimum* spp. extracts	Leaves, solvent extraction	In vitro	α-Amylase & α-glucosidase inhibition	α-Amylase IC50: 32.4 μg/mL; α-glucosidase IC50: 18.9 μg/mL	Modified CONSORT: 8/8
**Damic & Matejic (2022) [129]**	Serbia	Upper-middle income	Plant products including basil	Extracts	In vitro	DPP-IV inhibition assay	DPP-IV inhibition: 20–40% at 100 μg/mL	Modified CONSORT: 6/8
**Parasuraman et al. (2015) [124]**	India	Lower-middle income	*O. tenuiflorum* hydroalcoholic extract (250–500 mg/kg, 21 days)	Leaves, hydroalcoholic extraction	In vivo	STZ-induced diabetic rats	FBG reduction equivalent to glibenclamide	SYRCLE: moderate RoB
**Ogidi et al. (2024) [125]**	Nigeria	Lower-middle income	*O. tenuiflorum* ethanolic extract (400 mg/kg/day)	Leaves, ethanol extraction	In vivo	Diabetic albino rats	Comparable to metformin 70 mg/kg; reduced TC, TG, LDL; increased HDL	SYRCLE: moderate RoB
**Kanmaz et al. (2023) [133]**	Turkey	Upper-middle income	*O. basilicum* EO & aqueous extract	Leaves, hydrodistillation & aqueous extraction	In vivo	STZ-induced diabetic Wistar rats	FBG reduction + lipid profile data reported	SYRCLE: moderate RoB
**Oyewunmina**	Nigeria	Lower-middle income	Biherbal *O. gratissimum* formulation	Leaves, combined extract	In vivo	Induced diabetic albino rats	Blood glucose and lipid profile outcomes	SYRCLE: moderate-high RoB

Author	Country of Research	World Bank Income Classification (2024-2025)	Species / Preparation	Plant Material Used	Study Design	Organisms / Model / Cell Line	Key Quantitative Outcome	Methodological Quality Indicators[1]
**(2025) [130]**								
**Othman et al. (2021) [120]**	Saudi Arabia	High income	*O. basilicum* flavonoid fraction	Leaves, fractionation	In vivo	Diabetic albino rats	Hypoglycemic + antioxidant + anti-inflammatory effects quantified	SYRCLE: low-moderate RoB
**Perna et al. (2022) [29]**	Italy	High income	*Ocimum* spp. extracts/compounds	Various extracts & isolated compounds	In vitro + In vivo	HeLa, HepG2, MCF-7; Ehrlich ascites (mouse)	Linalool IC50: 75–150 µg/mL; rosmarinic acid IC50: 45–90 µM; caspase/Bax/Bcl-2 data	In vitro: 8/8; SYRCLE: moderate RoB
**Poulios et al. (2025) [33]**	Greece	High income	*O. basilicum*	Leaves, solvent extraction	In vitro	Multiple cancer cell lines	IC50 values; apoptosis, antioxidant, antimicrobial data	Modified CONSORT: 8/8
**Ayeda & Awda (2023) [135]**	Iraq	Lower-middle income	*O. basilicum* seed extracts	Seeds, solvent extraction	In vitro	Breast cancer cell lines	Cytotoxic IC50 values	Modified CONSORT: 6/8
**Alharbi (2024) [136]**	Saudi Arabia	High income	*O. forsskaolii* extract & EO	Aerial parts, solvent extraction & hydrodistillation	In vitro + In vivo	Cancer cell lines + CCl_4 hepatotoxicity (mice)	IC50 for anticancer, anti-inflammatory, antioxidant	In vitro: 7/8; SYRCLE: moderate RoB
**Kathirvel & Ravi (2012) [137]**	India	Lower-middle income	*O. basilicum* EO	Aerial parts, hydrodistillation	In vitro	HeLa, HEp-2, NIH 3T3 (fibroblasts)	IC50 for cytotoxicity; selective	Modified CONSORT: 7/8

Author	Country of Research	World Bank Income Classification (2024-2025)	Species / Preparation	Plant Material Used	Study Design	Organisms / Model / Cell Line	Key Quantitative Outcome	Methodological Quality Indicators¹
							neoplastic toxicity shown	
**Chen et al. (2017) [138]**	Taiwan	High income	Rosmarinic acid (isolated)	Isolated compound	In vitro	Pterygium epithelial cells	Apoptosis induction; IC50 quantified	Modified CONSORT: 8/8
**Ma et al. (2020) [139]**	China	Upper-middle income	Rosmarinic acid (isolated)	Isolated compound	In vitro	Osteosarcoma cells	Bax/Bcl-2; caspase-3; PTEN-PI3K-Akt pathway quantified	Modified CONSORT: 8/8
**Aldeeb et al. (2025) [142]**	Malaysia	Upper-middle income	*O. basilicum* ethanolic extract	Leaves, ethanol extraction	In vitro + Ex vivo	Endothelial cells (anti-angiogenic)	Endothelial inhibition quantified	Modified CONSORT: 7/8
**Nangia-Makker et al. (2013) [141]**	USA	High income	*O. gratissimum* extract	Leaves, solvent extraction	In vitro + In vivo	Breast cancer + MMP-2/MMP-9 assays	MMP inhibition; EMT reversal; VEGF suppression	In vitro: 8/8; SYRCLE: moderate RoB
**Sant'Ana et al. (2024) [143]**	Brazil	Upper-middle income	*O. basilicum* extract (100–200 mg/kg)	Leaves, solvent extraction	In vivo	Ehrlich ascites carcinoma (murine)	45–60% tumor volume reduction	SYRCLE: moderate RoB
**Touiss et al. (2019) [144]**	Morocco	Lower-middle income	Rosmarinic acid-rich O. basilicum extract	Leaves, fractionation	In vivo	High-fat diet hyperlipidemic mice	TC, TG, LDL reduction; lipid oxidation prevention	SYRCLE: moderate RoB
**Qamar et al. (2023) [118]**	Pakistan	Lower-middle income	*O. basilicum*	Leaves, solvent extraction	In vivo	L-NAME-induced hypertensive rats	SBP reduction: 15–25 mmHg	SYRCLE: moderate RoB

Author	Country of Research	World Bank Income Classification (2024-2025)	Species / Preparation	Plant Material Used	Study Design	Organisms / Model / Cell Line	Key Quantitative Outcome	Methodological Quality Indicators¹
**Suidah et al. (2026) [145]**	Indonesia	Upper-middle income	*O. basilicum* leaf extract	Leaves, solvent extraction	In vivo	Prednisone-treated hypertensive rats	VCAM-1 and eNOS modulation; BP reduction quantified	SYRCLE: moderate RoB
**Huang et al. (2024) [184]**	Taiwan	High income	Eugenol (isolated compound)	Isolated compound	In vitro + In vivo	Platelet activation + pulmonary thromboembolism (murine + human)	Platelet aggregation inhibition quantified	In vitro: 8/8; SYRCLE: moderate RoB
**Farag et al. (2016) [153]**	Egypt	Lower-middle income	EOs from four *Ocimum* spp.	Aerial parts, hydrodistillation	In vitro	AChE & BChE inhibition assays	IC50: 0.5–2 mg/mL for cholinesterase inhibition	Modified CONSORT: 7/8
**Siqueira et al. (2025) [152]**	Brazil	Upper-middle income	*O. americanum* extract	Leaves, solvent extraction	In vivo	Aging/neuroprotection model (rats)	Behavioral and biochemical neuroprotection data	SYRCLE: moderate RoB
**Amini-Khoei et al. (2025) [155]**	Iran	Upper-middle income	*O. basilicum* extract	Leaves, solvent extraction	In vivo	Maternal separation autism-like model (mice)	Neuroinflammation and oxidative stress markers quantified	SYRCLE: moderate RoB
**Ayuob et al. (2018) [156]**	Saudi Arabia	High income	*O. basilicum*	Leaves, solvent extraction	In vivo	Chronic stress neurodegeneration (mice hippocampus)	Histomorphometric and biochemical outcomes	SYRCLE: moderate RoB
**Dias et al. (2025) [158]**	Brazil	Upper-middle income	*O. basilicum* topical extract	Leaves, topical formulation	In vivo	Excisional wounds (mice)	Wound closure rate and histological data	SYRCLE: moderate RoB

Author	Country of Research	World Bank Income Classification (2024-2025)	Species / Preparation	Plant Material Used	Study Design	Organisms / Model / Cell Line	Key Quantitative Outcome	Methodological Quality Indicators¹
**Khan et al. (2020) [159]**	Pakistan	Lower-middle income	*O. basilicum* emulgel	Leaves, emulgel formulation	In vivo	Wound healing animal model	Tensile strength; wound contraction % reported	SYRCLE: moderate-high RoB
**Khalid et al. (2025) [160]**	India	Lower-middle income	*O. canum* extract	Whole plant, solvent extraction	In vitro + In vivo	Anti-inflammatory + wound healing	Bioassay-guided IC50 for anti-inflammatory activity	In vitro: 7/8; SYRCLE: moderate RoB
**Adtani et al. (2018) [162]**	India	Lower-middle income	*O. basilicum* & linalool	Leaves, extract & isolated compound	In vitro	Arecoline-induced fibrosis, buccal fibroblasts	Antifibrotic effects quantified	Modified CONSORT: 7/8
**Osorio (2011) [166]**	Philippines	Lower-middle income	*O. basilicum* EO (3% formulation)	Aerial parts, hydrodistillation	In vitro	Cutibacterium acnes	51 mm inhibition zone	Modified CONSORT: 5/8
**Ochola et al. (2022) [17]**	Kenya	Lower-middle income	*O. kilimandscharicum* EO formulation	Leaves, hydrodistillation	In vivo (field + lab)	*Anopheles* spp. larvae	LC50 and larval mortality % under field conditions	Field: moderate quality (confounding NR)
**Mahendran & Vimolmangkang (2023) [83]**	Thailand	Upper-middle income	*O. americanum* & *O. basilicum* EOs	Leaves, hydrodistillation	In vitro	Mosquito larvae	LC50 (larvicidal) + antimicrobial MIC	Modified CONSORT: 7/8
**Malima et al. (2013) [168]**	Tanzania	Low income	Tanzanian *Ocimum* spp. EOs	Aerial parts, hydrodistillation	In vivo (field)	*Anopheles* & Culex vectors	Repellence efficacy (%) quantified	Field: moderate quality
**Maryam & Alkali (2025) [169]**	Nigeria	Lower-middle income	*O. gratissimum* methanol extract	Leaves, methanol extraction	In vivo	*Anopheles* spp. larvae (Nigeria)	Larval mortality >90% at tested doses	Field: moderate quality

Author	Country of Research	World Bank Income Classification (2024-2025)	Species / Preparation	Plant Material Used	Study Design	Organisms / Model / Cell Line	Key Quantitative Outcome	Methodological Quality Indicators[1]
**Grellier (2014) [170]**	France/Mali	High income/Low income	*O. basilicum, O. canum,* Cymbopogon EOs	Aerial parts, hydrodistillation	In vitro + In vivo	*P. falciparum* + *Anopheles* funestus larvae	Antiplasmodial IC50 + larvicidal mortality data	Modified CONSORT: 7/8; Field: moderate
**Laraib et al. (2018) [167]**	Pakistan	Lower-middle income	*O. basilicum* extracts	Leaves, solvent extraction	In vitro	Culex quinquefasciatus larvae	Larvicidal LC50 values reported	Modified CONSORT: 6/8
**Teofilovic et al. (2021) [121]**	Serbia	Upper-middle income	*O. basilicum* extract	Aerial parts, solvent extraction	In vivo	Acetaminophen hepatotoxicity (rats)	ALT, AST normalization; hepatic antioxidant enzymes	SYRCLE: moderate RoB
**Abdullah et al. (2025) [174]**	Iraq	Lower-middle income	*O. basilicum* herb extract	Whole herb, solvent extraction	In vivo	Ethanol-induced liver damage (rats)	Pro-inflammatory cytokine levels reported	SYRCLE: moderate-high RoB
**Larbie et al. (2021) [175]**	Ghana	Lower-middle income	*O. americanum* extracts	Leaves, solvent extraction	In vivo	Gentamicin/cisplatin nephrotoxicity (rats)	Serum creatinine, urea, antioxidant enzyme data	SYRCLE: moderate RoB
**Thakur et al. (2025) [176]**	India	Lower-middle income	*O. tenuiflorum* hydroethanolic extract	Leaves, hydroethanolic extraction	In vivo	Gentamicin-induced AKI (rats)	Creatinine, oxidative/nitrosative stress markers	SYRCLE: moderate RoB
**Ben Mansour et al. (2024) [122]**	Tunisia	Lower-middle income	*O. basilicum* seed extract	Seeds, solvent extraction	In vivo	CCl_4 renal toxicity (rats)	Antioxidant + protective outcomes quantified	SYRCLE: moderate RoB

Author	Country of Research	World Bank Income Classification (2024-2025)	Species / Preparation	Plant Material Used	Study Design	Organisms / Model / Cell Line	Key Quantitative Outcome	Methodological Quality Indicators¹
**Lopresti et al. (2022) [20]**	Australia	High income	*O. tenuiflorum* extract (Holixer™, 125 mg twice daily)	Leaves, standardized extract	RCT (double-blind, placebo-controlled)	Adults experiencing stress (n=150)	Validated scales for stress, mood, sleep; effect sizes reported	Cochrane RoB 2: Some concerns (randomization process, missing outcome data)
**Abdi et al. (2025) [185]**	Iran	Upper-middle income	*O. basilicum*	Leaves, extract	RCT (triple-blind, placebo-controlled)	Postpartum women (n=120)	Postpartum depression prevention + sleep quality outcomes	Cochrane RoB 2: Some concerns
**Sestili et al. (2018) [178]**	Italy/ Pakistan	High income/Lower-middle income	*O. basilicum*	Aerial parts, extract	In vivo	Wistar rats (acute oral)	LD50 >5,000 mg/kg	OECD-compliant: acceptable
**Rasekh et al. (2012) [179]**	Iran	Upper-middle income	*O. basilicum* hydroalcoholic extract	Leaves, hydroalcoholic extraction	In vivo	Wistar rats (acute & subchronic)	LD50 and NOAEL values reported	OECD-compliant: acceptable
**Murugan et al. (2025) [181]**	India	Lower-middle income	*O. tenuiflorum* (Holixer™)	Leaves, standardized extract	In vivo	Genotoxicity + acute oral toxicity (rodents)	LD50 and genotoxicity endpoints	OECD-compliant: acceptable

Ethnobotanical Surveys

Author	Country of Research	World Bank Income Classification (2024-2025)	Species / Preparation	Plant Material Used	Study Design	Organisms / Model / Cell Line	Key Quantitative Outcome	Methodological Quality Indicators¹

Author	Country of Research	World Bank Income Classification (2024-2025)	Species / Preparation	Plant Material Used	Study Design	Organisms / Model / Cell Line	Key Quantitative Outcome	Methodological Quality Indicators[1]
**Shikha & Kashyap (2023) [9]**	India	Lower-middle income	*O. basilicum*, *O. tenuiflorum*, *O. gratissimum*, *O. americanum*, *O. kilimandscharicum*	Leaves, whole plant, aerial parts	Cross-sectional ethnobotanical survey	Traditional healers and rural households (n=210)	Use reports: gastrointestinal (82%), respiratory (76%), fever/malaria (65%), skin infections (59%), diabetes (41%)	JBI: 7/9 (sample size justification NR, validity of instrument NR)
**Induar et al. (2024) [10]**	India	Lower-middle income	*O. basilicum*, *O. tenuiflorum*, *O. gratissimum*, *O. americanum*, *O. canum*	Leaves, seeds, whole plant	primary survey	Traditional medicine practitioners (n=85)	Fidelity level (FL): *O. tenuiflorum* for respiratory ailments (FL=0.89); *O. gratissimum* for wounds (FL=0.82)	JBI: 6/9 (sampling method unclear, recall bias possible)
**Jahanger et al. (2023) [44]**	India	Lower-middle income	*O. basilicum*, *O. tenuiflorum*, *O. gratissimum*, *O. americanum*	Leaves, whole plant	Ethnopharmacological survey	Rural communities in Uttar Pradesh (n=156)	Use value (UV): *O. tenuiflorum* UV=0.92; *O. gratissimum* UV=0.78; informant consensus factor (ICF)=0.85 for digestive disorders	JBI: 8/9 (validated questionnaire, systematic sampling)

Author	Country of Research	World Bank Income Classification (2024-2025)	Species / Preparation	Plant Material Used	Study Design	Organisms / Model / Cell Line	Key Quantitative Outcome	Methodological Quality Indicators¹
**Ugbogu et al. (2021) [48]**	Nigeria	Lower-middle income	*O. gratissimum* (clove basil)	Leaves, essential oil, aqueous extract	Ethnobotanical survey	Traditional healers in southeastern Nigeria (n=120)	Frequency of citation: antidiabetic (67%), antimalarial (58%), antimicrobial (72%), wound healing (63%)	JBI: 7/9 (adequate sample size, but recall bias possible)
**Babandi (2025) [26]**	Nigeria	Lower-middle income	Multiple *Ocimum* spp. including *O. gratissimum*, *O. basilicum*	Leaves, decoctions, macerations	Cross-sectional ethnomedicinal survey	Rural communities in northern Nigeria (n=245)	Percentage of respondents reporting use: malaria/fever (78%), diarrhea (71%), respiratory infections (65%), skin infections (59%)	JBI: 8/9 (stratified sampling, validated instrument)
**Mazibuko et al. (2024) [50]**	Malawi	Low income	*O. canum* (Malawi camphor basil), *O. basilicum*	Leaves, steam inhalation, topical ointment	Ethnobotanical survey of emerging spices and herbs	Rural households and market vendors (n=180)	*O. canum* ranked as most effective mosquito repellent (mean score 4.2/5); use reported by 73% of respondents for respiratory complaints	JBI: 6/9 (convenience sampling, limited geographic scope)
**Oloya et al. (2025) [89]**	Uganda	Low income	*O. gratissimum*, *O. americanum*, *O. basilicum*	Leaves, smoke, topical application	Cross-sectional survey of	West Nile Subregion	*Ocimum* species used by 68% of respondents;	JBI: 7/9 (large sample size, but outcome not objectively measured)

Author	Country of Research	World Bank Income Classification (2024-2025)	Species / Preparation	Plant Material Used	Study Design	Organisms / Model / Cell Line	Key Quantitative Outcome	Methodological Quality Indicators¹
					mosquito repellent plants	households (n=400)	repellency efficacy self-reported as "highly effective" (52%) and "moderately effective" (38%)	
**Zahran et al. (2020) [46]**	Egypt	Lower-middle income	*O. basilicum*, *O. tenuiflorum*, *O. gratissimum*, *O. americanum*, *O. kilimandscharicum*	Leaves, whole plant	Ethnobotanical review with primary validation	Sinai Peninsula traditional healers (n=65)	Use reports: digestive disorders (74%), respiratory (68%), hypertension (52%), diabetes (45%); relative frequency of citation (RFC) values reported	JBI: 8/9 (systematic sampling, clear inclusion criteria)
**Prasongdee et al. (2024) [190]**	Thailand	Upper-middle income	*O. basilicum* (Thai basil), *O. tenuiflorum*	Leaves, whole plant	Ethnobotanical survey + phytochemical validation	Northern Thailand traditional medicine practitioners (n=95)	Citation frequency: antidiabetic (71%), flatulence/dyspepsia (68%), postnatal tonic (54%), dysmenorrhea (47%); cultural importance index (CI) values reported	JBI: 8/9 (snowball sampling limitation acknowledged, validated questionnaire)

Notes:

[1] **Methodological Quality Indicators:**

- Modified CONSORT for in vitro studies: Score out of 8 domains (positive/negative controls, replication ≥3, blinding of outcome assessment, dose-response reporting, sample size justification, statistical methods, control for batch effects, declaration of conflicts). Scores ≥7 = high quality; 5–6 = moderate; <5 = low.
- SYRCLE RoB for in vivo animal studies: Classification: low risk of bias, moderate risk, high risk, or unclear. Assessed across 10 domains.
- Cochrane RoB 2 for clinical trials: Classification: low risk, some concerns, or high risk.
- Field trials (vector control): Qualitative assessment: acceptable, moderate concerns, or high concerns based on site selection, randomization, blinding, loss to follow-up, environmental confounding.
- OECD-compliant toxicology: Acceptable if followed OECD guidelines 423 (acute) or 408 (sub chronic).

AChE: acetylcholinesterase; AKI: acute kidney injury; ALT: alanine aminotransferase; AST: aspartate aminotransferase; BChE: butyrylcholinesterase; BP: blood pressure; CCl_4: carbon tetrachloride; COX: cyclooxygenase; DPPH: 2,2-diphenyl-1-picrylhydrazyl; DPP-IV: dipeptidyl peptidase-IV; EMT: epithelial-mesenchymal transition; eNOS: endothelial nitric oxide synthase; EO: essential oil; FBG: fasting blood glucose; GPx: glutathione peroxidase; HDL: high-density lipoprotein; IC_{50}: half-maximal inhibitory concentration; IL-1β: interleukin-1 beta; IL-6: interleukin-6; LC_{50}: median lethal concentration; LD_{50}: median lethal dose; LDL: low-density lipoprotein; L-NAME: Nω-nitro-L-arginine methyl ester; LOX: lipoxygenase; LPS: lipopolysaccharide; MDA: malondialdehyde; MDR: multidrug-resistant; MIC: minimum inhibitory concentration; MMP: matrix metalloproteinase; MRSA: methicillin-resistant Staphylococcus aureus; n: sample size; NOAEL: no observed adverse effect level; NP: nanoparticle; NR: not reported; OECD: Organization for Economic Co-operation and Development; PTEN: phosphatase and tensin homolog; RCT: randomized controlled trial; RoB: risk of bias; SBP: systolic blood pressure; SOD: superoxide dismutase; STZ: streptozotocin; TC: total cholesterol; TFC: total flavonoid content; TG: triglycerides; TNF-α: tumor necrosis factor-alpha; TPC:, total phenolic content; VCAM-1: vascular cell adhesion protein-1; VEGF: vascular endothelial growth factor.

Quality assessment tools: CONSORT: Consolidated Standards of Reporting Trials; JBI: Joanna Briggs Institute; SYRCLE: Systematic Review Centre for Laboratory Animal Experimentation.
